# Met246 and Asn250 in the D2 protein are essential for the operation of the quinone-Fe-acceptor complex of Photosystem II

**DOI:** 10.1093/pcp/pcaf078

**Published:** 2025-07-14

**Authors:** Victor Zhong, Imre Vass, Priyanka Pradeep Patil, Julian J Eaton-Rye

**Affiliations:** Department of Biochemistry, University of Otago, P.O. Box 56, Dunedin 9054, New Zealand; HUN-REN, Biological Research Center, Institute of Plant Biology, P.O. Box 521, Temesvári krt. 62, Szeged H-6726, Hungary; HUN-REN, Biological Research Center, Institute of Plant Biology, P.O. Box 521, Temesvári krt. 62, Szeged H-6726, Hungary; Faculty of Science and Informatics, Doctoral School of Biology, University of Szeged, P.O. Box 652, Dóm tér 10, Szeged H-6720, Hungary; Department of Biochemistry, University of Otago, P.O. Box 56, Dunedin 9054, New Zealand

**Keywords:** bicarbonate, D2, Photosystem II, Q_A_, Q_B_, *Synechocystis* sp. PCC 6803

## Abstract

The chemical properties of the primary (Q_A_) and secondary (Q_B_) plastoquinone electron acceptors of Photosystem II (PS II) depend on their protein environments. The DE loop of the D2 protein (residues 222–262) contributes to the Q_A_-binding site while the DE loop of the D1 protein (residues 233–266) contributes to the Q_B_-binding environment. The roles of the invariant D2-Met246 and D2-Asn250 residues in the vicinity of the Q_A_-binding site have been investigated in the cyanobacterium *Synechocystis* sp. PCC 6803 using mutants targeting both residues. The M246F strain was phenotypically similar to control cells; however, the M246A, N250A, and N250H strains had slowed photoautotrophic growth and were sensitive to high light and the addition of formate. In addition, the M246K and N250N strains were unable to assemble PS II. Chlorophyll *a* fluorescence measurements indicated electron transfer between Q_A_ and Q_B_ was modified in the M246A, N250A, and N250H strains, and the exchange of plastoquinol between the Q_B_-binding site and the plastoquinone pool in the thylakoid membrane was impaired. Modified electron transfer in these mutants in the presence or absence of formate was restored by the addition of bicarbonate. In addition, thermoluminescence measurements showed a down shift in the redox midpoint potential of the Q_A_/Q_A_^**−**^ couple in the N250A and N250H strains. These results demonstrate that Met246 and Asn250 play indispensable roles in the quinone-iron-acceptor complex, influencing both Q_A_ binding and the binding of the bicarbonate ligand to the non-heme iron that is located between Q_A_ and Q_B_.

## Introduction

Photosystem II (PS II) in plants and cyanobacteria catalyzes the light-driven splitting of water in oxygenic photosynthesis (Shen, 2015; Vinyard and Brudvig, 2017; Shen et al., 2021). The use of cyanobacteria as model systems to study PS II has benefitted from several high-resolution structures obtained by X-ray-crystallography and cryo-electron microscopy (Umena et al., 2011; Kern et al., 2018; Gisriel et al., 2022; Hussein et al., 2024). Cyanobacterial PS II is a dimeric membrane-spanning complex of ~700 kDa, with each monomer consisting of ~20 subunits. The reaction center subunits D1 and D2 bind most of the redox-active cofactors with D1 providing ligands for the Mn_4_CaO_5_ oxygen-evolving complex (OEC). The D1 and D2 polypeptides are flanked by the chlorophyll *a*-binding core antenna subunits CP43 and CP47, with CP43 also contributing to the binding environment of the OEC (Ferreira et al., 2004; Shen, 2015). The CP43-D1-D2-CP47 core is surrounded by 13 low-molecular-weight (LMW) proteins and three or four additional extrinsic proteins are present that stabilize the OEC and participate in the access of substrate water as well as the egress of protons and oxygen (Shen, 2015; Gisriel et al., 2022).

Biogenesis of PS II proceeds in a stepwise manner with the initial formation of a reaction center complex from two pre-formed D1 and D2 modules (Nickelsen and Rengstl, 2013). A CP47 module then associates to form the intermediate RC47 complex. Subsequently, a CP43 module binds to the RC47 complex followed by light-driven assembly of the OEC, the binding of the extrinsic subunits, and subsequent dimerization (Zabret et al., 2021; Komenda et al., 2024). Each of the four assembly modules contain specific LMW subunits and assembly factors, but the assembly factors are not retained in the mature photosystem (Komenda et al., 2024). In the final step, the mature dimer goes on to form a complex with the peripheral light-harvesting phycobilisome. Photochemistry and water-splitting by PS II, however, results in the generation of reactive oxygen species (ROS) and in oxidative damage such that PS II requires continuous repair (Adir et al., 2003; Vass, 2012; Pospíšil, 2016). Under stress conditions, photoinhibition occurs if light-induced photodamage exceeds the capacity of the repair process (Nishiyama et al., 2011; Johnson and Pakrasi, 2022).

In PS II, the absorbed light energy facilitates the transfer of electrons from water to plastoquinone via the reaction center chlorophylls that make up P680 (Cardona et al., 2012). Upon excitation, P680^*^ reduces pheophytin, which, in turn, reduces the primary plastoquinone electron acceptor, Q_A_ (Cardona et al., 2012). Q_A_ is a one-electron carrier that is bound to the D2 protein. The electron on Q_A_ is then transferred to the secondary plastoquinone electron acceptor, Q_B_, which is bound to the D1 protein. The secondary quinone acceptor, Q_B_, undergoes two reduction steps accompanied by protonation leading to the formation of plastoquinol (Q_B_H_2_ or PQH_2_) (Müh et al., 2012). Following this, PQH_2_ dissociates from the Q_B_-binding site and is replaced by an oxidized plastoquinone (PQ) from a PQ pool in the thylakoid membrane (Van Eerden et al., 2017). The oxidized primary donor is then reduced by electrons from the OEC, which transitions through a series of oxidation states denoted as the S-states (ranging from S_0_ to S_4_) resulting in the formation of oxygen by splitting two water molecules after four successive turnovers of the reaction center (Kok et al., 1970; Bhowmick et al., 2023; Li et al., 2024).

A non-heme iron (NHI) is situated between Q_A_ and Q_B_ on the acceptor side of PS II. The NHI is not redox active and is coordinated by two D1 histidine residues (His215 and His272), two D2 histidine residues (His214 and His268), and a bidentate bicarbonate (HCO_3_^−^) ligand (Umena et al., 2011; Shevela et al., 2012; Tamura et al., 2021). The absence of bicarbonate disrupts forward electron transfer between Q_A_ and Q_B_ and impairs the PQH_2_/PQ exchange reactions at the Q_B_-binding site (Eaton-Rye and Govindjee 1988a; Eaton-Rye and Govindjee 1988b; Sedoud et al., 2011). Furthermore, a role in photoprotection has been suggested for the bicarbonate ligand (Brinkert et al., 2016; Sugo and Ishikita, 2022). Under photoinhibitory conditions, prolonged reduction of Q_A_^**−**^ may lead to the dissociation or reorientation of the bicarbonate ligand from the NHI (Brinkert et al., 2016; Sugo and Ishikita, 2022). This perturbation induces a positive shift in the redox potential of the Q_A_/Q_A_^**−**^ couple, promoting a back reaction with P680^+^ that prevents the production of singlet oxygen (Brinkert et al., 2016; Kato and Noguchi, 2021; Fantuzzi et al., 2022; Sugo and Ishikita, 2022).

**Figure 1:**
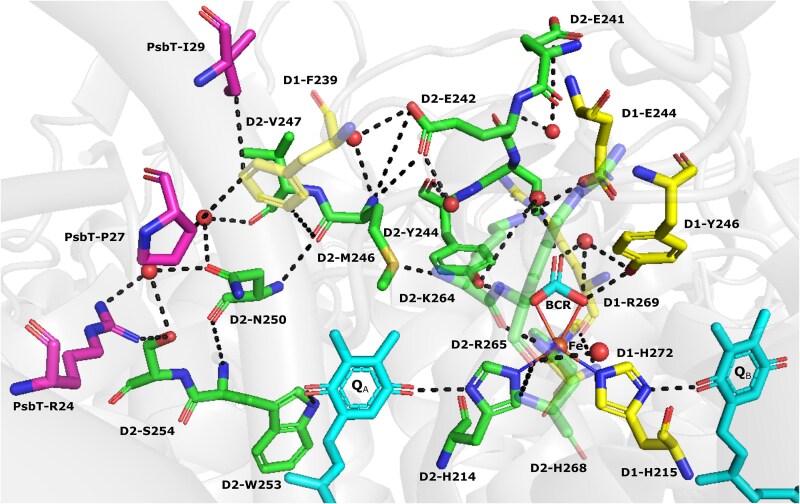
Diagram of the PS II acceptor side hydrogen-bond network around the non-heme iron and bicarbonate-binding environment. BCR represents bicarbonate, Fe is the non-heme iron and red spheres indicate water molecules. Q_A_ and Q_B_ are shown in light blue. Oxygen atoms are shown in red, nitrogen atoms are shown in blue, and the sulfur atom in Met246 is shown in yellow. D1 residues are in yellow, D2 residues are in green, and PsbT residues are in magenta. Dashed black lines indicate putative hydrogen bonds. The figure was generated using PyMOL and PDB 7N8O.

The DE loop, connecting the transmembrane helices D and E of the D2 protein, is highly conserved across oxygenic organisms (Kless et al., 1992 and Supplementary Fig. S1). Specifically, the sequence ^240^A**EETY**SMVTAN^250^ within the DE loop of the D2 protein of PS II represents a conserved motif surrounding the bicarbonate ligand to the NHI (Khaing et al., 2022; Khaing and Eaton-Rye, 2023). This conserved motif is also present in the D1 DE loop, ^242^E**EETY**NIVAAH^252^, with both loops containing a conserved EETY sequence and mutations targeting this sequence disrupt both Q_A_ to Q_B_ electron transfer and PS II assembly (Cardona et al., 2015; Forsman et al., 2019; Khaing et al., 2022). In this study, the roles of D2-Met246 and D2-Asn250, which are in the proximity of the Q_A_ binding site (Fig. 1), were investigated by introducing mutations in the cyanobacterium *Synechocystis* sp. PCC 6803 (hereafter *Synechocystis* 6803). Our findings indicate that substitutions within the D2 DE loop at Met246 and Asn250 disrupt Q_A_ and bicarbonate binding, resulting in impaired assembly and function of PS II.

## Results

### Photoautotrophic growth and PS II assembly

**Figure 2:**
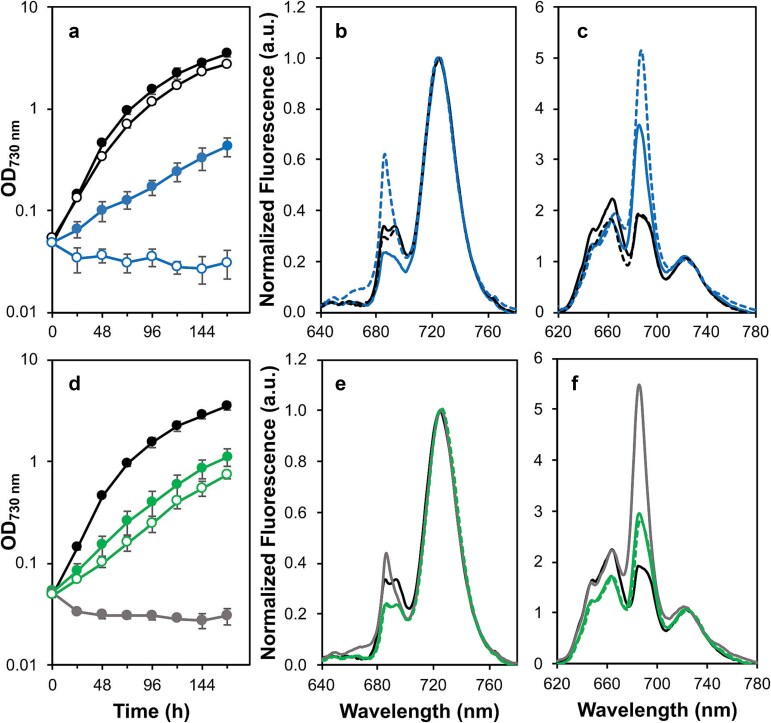
Photoautotrophic growth and low-temperature (77 K) fluorescence emission spectra. (a) Photoautotrophic growth measured by turbidity at 730 nm. Control (black filled circles), M246A (blue filled circles), M246F (black empty circles) and M246K (blue empty circles). Data are the average of three independent experiments and the standard error is shown. (b) Low-temperature emission spectra from cells excited at 440 nm. Control (black line), M246A (blue line), M246F (black dashed line) and M246K (blue dashed line). (c) Low-temperature emission spectra from cells excited at 580 nm. Strains are the same as panel b. (d) Photoautotrophic growth measured by turbidity at 730 nm. Control (black filled circles), N250A (green filled circles), N250D (gray filled circles), and N250H (green empty circles). Data are the average of three independent experiments and the standard error is shown. (e) Low-temperature emission spectra from cells excited at 440 nm. Control (black line), N250A (green line), N250D (gray line) and N250H (green dashed line line). (f) Low-temperature emission spectra from cells excited at 580 nm. Strains are the same as panel e. The low-temperature emission spectra are the average of three independent experiments and normalized to the PS I peak at 725 nm.

To investigate the role of Met246 in the DE-loop of D2, the M246A, M246F, and M246K mutants were constructed (see Materials and Methods). The impact of these introduced changes was initially evaluated by measuring photoautotrophic growth in combination with low-temperature (77 K) fluorescence emission spectroscopy to assess the ability of the mutants to assemble PS II. The M246F strain (doubling time, 18 h) exhibited a photoautotrophic growth rate similar to the control (doubling time, 15 h). In contrast, the M246A strain exhibited impaired photoautotrophic growth with a doubling time of 46 h, while the M246K mutant was an obligate photoheterotroph (Fig. 2a).

Low-temperature fluorescence emission spectra resulting from excitation at 440 nm exhibit peaks at 685 and 695 nm from PS II and 725 nm from PS I (Lamb et al., 2018). The 685 nm emission arises from PS II pre-assembly complexes and CP43 in assembled PS II while the 695 nm emission comes from CP47 in assembled complexes (Boehm et al., 2011). Under 440 nm excitation, the M246F strain had a PS II to PS I ratio similar to that observed in control cells; however, the M246A strain exhibited a reduction in fluorescence yield at 685 nm and 695 nm, indicating a potential decrease in this ratio with the lower 695 nm peak suggesting fewer assembled PS II centers. In contrast, the M246K strain displayed an increased fluorescence yield at 685 nm, but lacked the 695 nm peak, implying the absence of assembled PS II centers along with an accumulation of intermediate complexes (Fig. 2b). When fluorescence emission was measured following excitation at 580 nm, the M246F strain displayed characteristics similar to the control, whereas both the M246A and M246K mutants exhibited a prominent emission at 685 nm, suggesting a potential uncoupling of the phycobilisome and impaired energy transfer to PS II due to a reduction or absence of assembled PS II centers (Fig. 2c).

A similar approach was taken to initially evaluate the introduction of mutations at Asn250 in the DE-loop. The N250A and N250H strains demonstrated reduced photoautotrophic growth rates with doubling times of 32 h and 45 h, respectively, while the N250D strain was not able to grow photoautotrophically (Fig. 2d). The N250A and N250H strains also displayed a reduced yield in their low-temperature fluorescence emission spectra at 685 nm and 695 nm following excitation at 440 nm, consistent with fewer assembled PS II centers. The N250D strain, however, showed an increased 685 nm peak while lacking the 695 nm peak indicative of reduced assembly of PS II (Fig. 2e). The corresponding fluorescence emission following excitation at 580 nm revealed both the N250A and N250H strains to have an increased emission at 685 nm with the N250D strain exhibiting a larger emission at 685 nm, consistent with the apparent reduction of assembled PS II in these strains (Fig. 2f).

**Figure 3:**
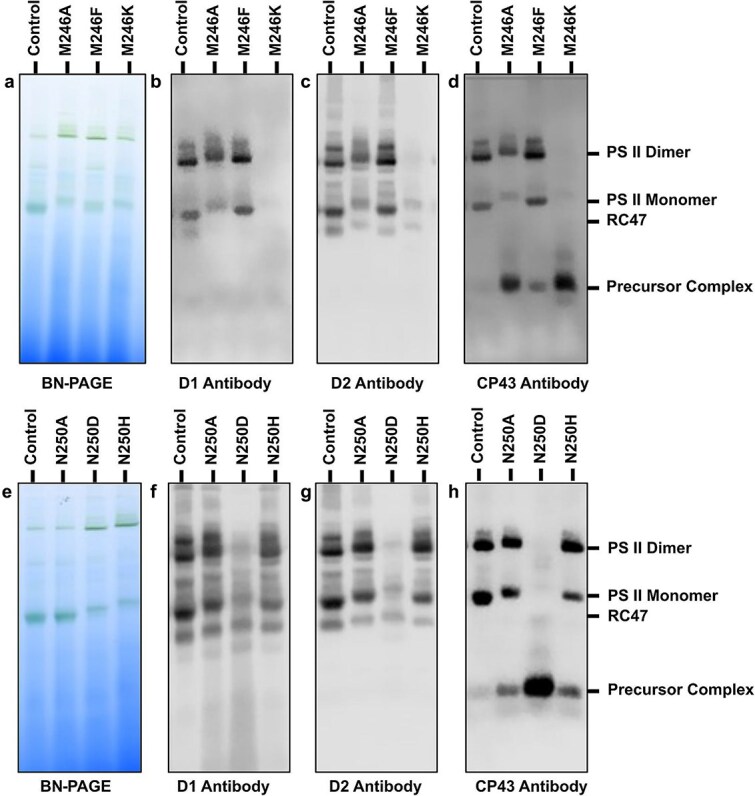
Blue-native (BN) PAGE followed by western blot analysis. Panels a-d are for strains with mutations at Met246 and panels e-h are for strains with mutations at Asn250. (a, e) BN-PAGE gel. (b, f) western blot with antibody against D1. (c, g) western blot with antibody against D2. (d, h) western blot with antibody against CP43. The strains are labeled above their corresponding lane. RC47 is an assembly intermediate of PS II that lacks bound CP43.

To independently assess the extent of PS II assembly in the different strains, BN-PAGE followed by western blotting using PS II-specific antibodies was performed (Fig. 3). In the M246F strain the level of PS II monomers and dimers resembled those of the control, but an accumulation of unassembled pre-assembly complexes or modules containing CP43 was evident. The M246A mutant exhibited reduced PS II dimer levels and a notable decrease in PS II monomers. Moreover, there was a substantial buildup of unassembled pre-assembly complexes or modules containing CP43 and a mobility shift in protein complexes was observed, possibly indicating stress-induced carotenoid production (Choo et al., 2021). The M246K mutant showed minimal PS II assembly, with faint bands detected in the α-D2 western blot, which may suggest trace PS II amounts. Like the M246A mutant, a substantial accumulation of unassembled pre-assembly complexes or modules containing CP43 was also observed in the thylakoids of the M246K strain (Fig. 3a–d).

Both the N250A and N250H strains exhibited reduced PS II monomer levels and the presence of CP43-containing unassembled pre-assembly complexes or modules. In addition, the N250D mutant only showed trace amounts of PS II dimers and monomers, alongside a pronounced accumulation of unassembled CP43. A shift in protein complex mobility was also observed, suggesting potential stress-induced carotenoid production (Fig. 3e–h). The presence of unassembled pre-assembly complexes or modules containing CP43 suggests a disruption in the incorporation of CP43 into the RC47 complex during assembly or impaired repair in the various mutants.

### Steady-state PS II activity in mutants with substitutions at Met246

**Figure 4:**
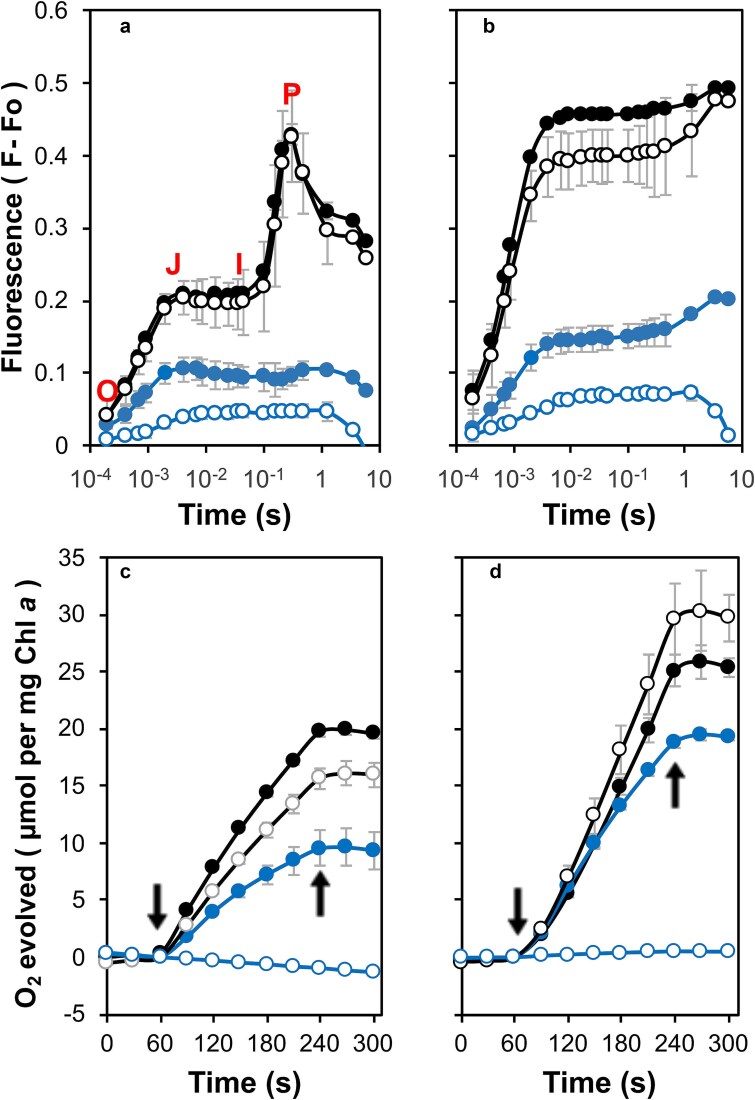
Photosystem II activity in the strains with mutations at Met246. (a) Variable chlorophyll *a* fluorescence in the absence of DCMU. Control (black filled circles), M246A (blue filled circles), M246F (black empty circles) and M246K (blue empty circles). O is the origin, J and I inflections and P the peak of the observed fluorescence in the control. (b) Variable chlorophyll *a* fluorescence in the presence of DCMU. Strains are the same as shown in panel a. (c) Oxygen evolution supported by 200 μM DMBQ and 1 mM K_3_Fe(CN)_6_. Arrows indicate light on and off. Strains are the same as shown in panel a. (d) Oxygen evolution supported by 15 mM bicarbonate. Arrows indicate light on and off. Strains are the same as shown in panel a. Data are the average of three biological repeats.

Variable chlorophyll fluorescence induction assays were carried out to evaluate electron transfer in the mutant strains. In the absence of 3,4-dichloro-1,1-dimethyl urea (DCMU), the control strain displayed a typical OJIP induction curve, where the initial rise from F_o_ or ‘O’ to ‘J’ arises due to photochemical reduction of Q_A_, and this is followed by an increase to ‘I’ followed by a maximum F_m_ or ‘P’ as the downstream electron acceptors become reduced (Fig. 4a). Subsequently, fluorescence declined due to Calvin-Benson cycle activation and various quenching mechanisms (Stirbet and Govindjee, 2011). Upon the addition of DCMU, fluorescence promptly reached Fm, attributed to the inhibition of forward electron transfer from Q_A_^−^ (Fig. 4b). The variable fluorescence yield in the presence of DCMU typically reflects the relative level of active PS II centers (Vass et al., 1999). In addition, the rise observed following 1 s of actinic illumination reflects a State 2 (energy preferentially directed to PS I) to State 1 (energy preferentially directed to PS II) transition (Kaňa et al., 2012; Calzadilla and Kirilovsky, 2020). In the absence of DCMU, the M246F mutant displayed kinetics similar to the control strain, but a slight decrease in F_m_ was apparent upon the addition of DCMU. In contrast, the M246A mutant showed a marked reduction in F_m_ both in the presence and absence of DCMU and this was more pronounced in the M246K mutant.

PS II activity was also assessed through oxygen evolution assays conducted in the presence of 2,5-dimethyl-1,4-benzoquinone (DMBQ) and sodium bicarbonate (Fig. 4c, d). In the presence of DMBQ, the rates of oxygen evolution in the M246A and M246F strains were reduced to 51% and 76% of the control strain, respectively (Fig. 4c and Supplementary Table S1). However, in the presence of bicarbonate, the oxygen evolution rate in the M246F strain slightly exceeded the control at 107%, while the oxygen evolution rate of the M246A strain improved to 91% of the control rate (Fig. 4d and Supplementary Table S1). The M246K strain did not evolve oxygen in the presence of DMBQ; however, in the presence of bicarbonate, trace amounts of oxygen were observed.

### Steady-state PS II activity in mutants with substitutions at Asn250

**Figure 5:**
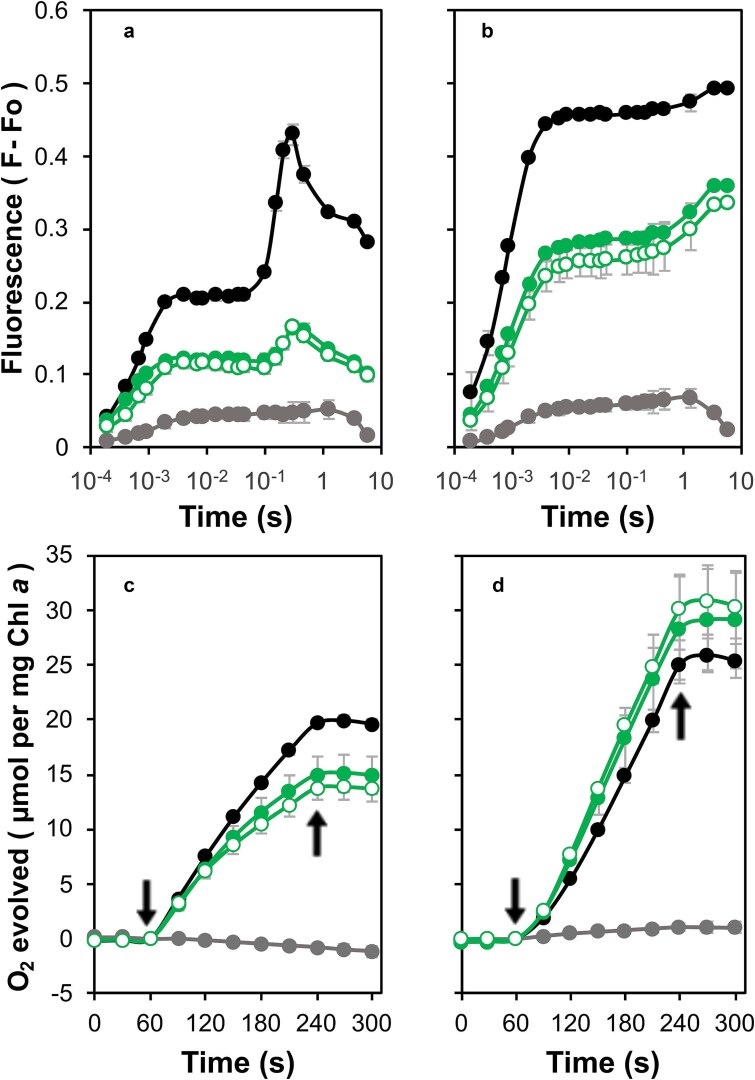
Photosystem II activity in the strains with mutations at Asn250. (a) Variable chlorophyll *a* fluorescence in the absence of DCMU. Control (black filled circles), N250A (green filled circles), N250D (gray filled circles) and N250H (green empty circles). (b) Variable chlorophyll *a* fluorescence in the presence of DCMU. Strains are the same as shown in panel a. (c) Oxygen evolution supported by 200 μM DMBQ and 1 mM K_3_Fe(CN)_6_. Arrows indicate light on and off. Strains are the same as shown in panel a (d) Oxygen evolution supported by 15 mM bicarbonate. Arrows indicate light on and off. Strains are the same as shown in panel a. Data are the average of three biological repeats.

Both the N250A and N250H strains also exhibited a reduced F_m_ relative to the control indicating fewer assembled or active PS II centers but in these mutants a small I to P rise was retained in the absence of DCMU (Fig. 5a, b). The N250D strain, however, showed almost no variable fluorescence consistent with the corresponding low-temperature emission spectra and BN-PAGE analysis. Oxygen evolution was also investigated in these mutants. In the presence of DMBQ, oxygen evolution by the N250A and N250H strains was reduced to 86% and 89% of the control rate, respectively (Fig. 5c and Supplementary Table S2). However, upon bicarbonate supplementation, both the N250A (111%) and N250H (121%) strains exhibited oxygen evolution rates surpassing those of the control, a phenomenon that has been observed in D2 strains carrying mutations at Arg24 and Arg26 (Biswas et al., 2024). In these assays, the N250D strain did not evolve oxygen in the presence of DMBQ; however, when bicarbonate was introduced, trace amounts of oxygen were detected (Fig. 5d and Supplementary Table S2).

### Impaired PS II activity under high light

**Figure 6:**
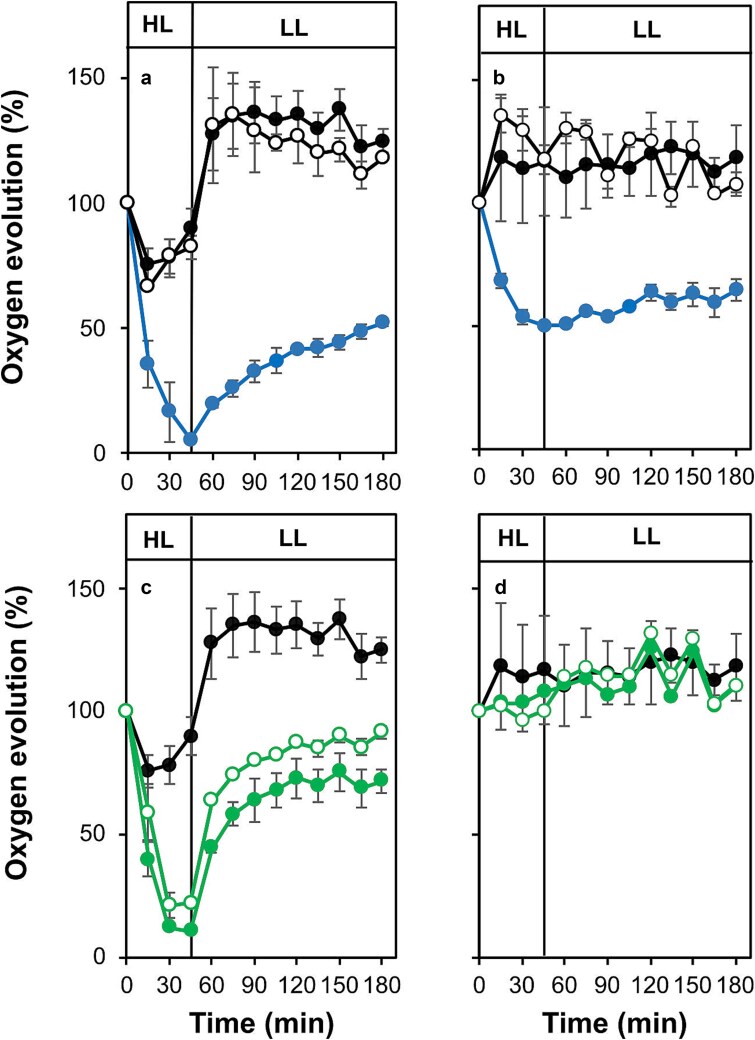
Sensitivity of oxygen evolution to high light (or photodamage) in control and mutant cells. (a) Photodamage in the presence of DMBQ and K_3_Fe(CN)_6_: Control (black filled circles), M246A (blue filled circles) and M246F (black empty circles). (b) Photodamage in the presence of HCO_3_^**−**^: Symbols as in panel a. (c) Photodamage in the presence of DMBQ and K_3_Fe(CN)_6_: Control (black filled circles), N250A (green filled circles) and N250H (green empty circles). (d) Photodamage in the presence of HCO_3_^**−**^: Symbols as in panel c. In all panels, high light (HL) was 2000 μmol photons m^−2^ s^−1^ (for 45 min) and low light (LL) 30 μmol photons m^−2^ s^−1^ (for 135 min). Error bars represent the standard error from three independent experiments. The control data are repeated in panels c and d to facilitate comparison between the different mutants.

Sensitivity towards high light was investigated to assess the susceptibility to photoinhibition and capacity for recovery in the different mutants. High light (2000 μmol photons m^−2^ s^−1^) was applied for 45 min followed by recovery under low light (30 μmol photons m^−2^ s^−1^) and PS II-specific oxygen evolution was measured in the presence of DMBQ (Fig. 6). The M246F strain behaved similarly to control cells adapting to the exposure to high light and exhibiting an elevated rate of oxygen evolution upon the transition to low light. In contrast, the M246A strain underwent rapid photodamage during the high-light treatment with an almost complete loss of PS II activity after 45 min followed by a limited recovery to only 52% of its initial rate after 135 min in low light (Fig. 6a). When assessing whole chain electron transfer in the presence of bicarbonate, oxygen evolution in control and M246F cells remained relatively constant throughout the assay; however, oxygen evolution in the M246A mutant dropped to 50% of its initial rate after 45 min in high light and did not recover (Fig. 6b).

Identical experiments were then performed on the N250A and N250H mutants. In the presence of DMBQ, oxygen evolution by the N250A strain rapidly dropped in response to the high-light treatment falling to 11% of its initial value after 45 min in high light. Likewise, the high-light treatment reduced the rate of oxygen evolution in N250H cells to 22% of their initial rate. Upon the transition to low light both strains exhibited a recovery with the N250A mutant reaching 75% and the N250H strain 92% of their initial rates over a period of 135 min (Fig. 6c). In contrast, both the N250A and N250H mutants behaved similarly to the control when oxygen evolution was assayed in the presence of bicarbonate (Fig. 6d). The results obtained in the presence of bicarbonate with N250A and N250H cells suggests, when whole chain electron transfer is measured in saturating light, there is sufficient turnover of PS II in the presence of the native quinone to result in the mutant cells supporting whole chain rates of electron transfer that are similar to those achieved by the control cells.

### Decay of variable chlorophyll *a* fluorescence and thermoluminescence in mutants with substitutions at Met246

**Figure 7:**
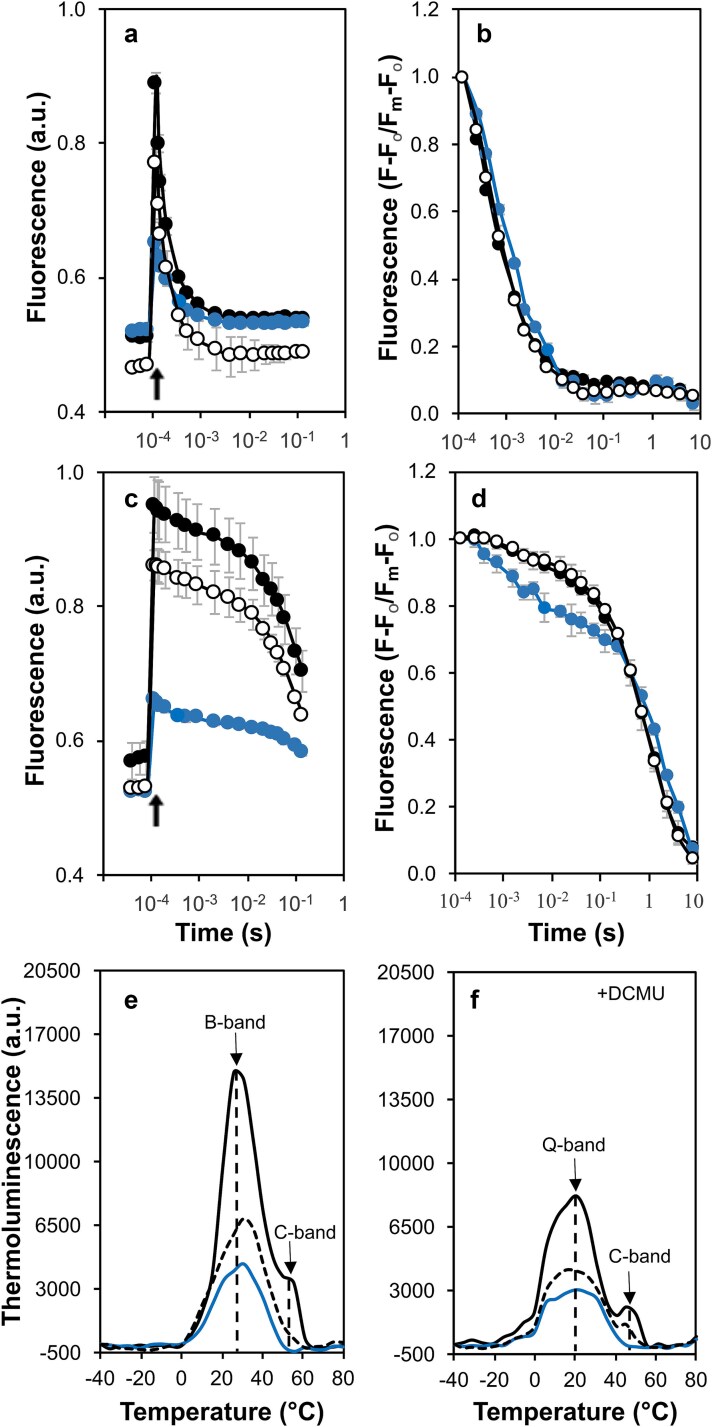
Chlorophyll *a* fluorescence decay after a single turnover actinic flash and thermoluminescence emission in the strains with mutations at Met246. (a) Fluorescence decay after three measurements determining the initial (or F_o_) fluorescence. Control (black filled circles), M246A (blue filled circles) and M246F (black empty circles). (b) Fluorescence decays from panel a normalized to the maximum fluorescence (Fm) where F is the fluorescence at each time point after the actinic flash. (c) Fluorescence decay after three measurements determining F_o_ in the presence of DCMU. Strains and symbols are the same as panel a. (d) Fluorescence decays from panel c normalized to Fm. In panels a–d, data are the average of three independent experiments. (e) Thermoluminescence emission in the absence of DCMU. Control (black line), M246A (blue line) and M246F (black dashed lines). (f) Thermoluminescence emission in the presence of DCMU. Strains are the same as panel e. In panels e and f, the data are the average of three independent biological repeats. All thermoluminescence curves were deconvoluted (Supplementary Fig. S2) and the temperature maxima of the individual peaks are listed in Table 2.

Measuring the decay of chlorophyll *a* fluorescence after a single actinic flash provides information regarding electron transfer between Q_A_ and Q_B_. The fluorescence decay kinetics in the absence of DCMU can be classified into three distinct phases: a fast (μs) phase indicative of Q_A_^−^ oxidation by bound Q_B_ or Q_B_^−^; an intermediate (ms) phase resulting from Q_B_ binding to an empty pocket and subsequent Q_A_^**−**^ oxidation, and a slow (s) phase corresponding to the back reaction Q_A_^−^ with the S_2_ state of the OEC (Vass et al., 1999). When subjected to a single actinic flash, the control strain exhibited a fast phase (t_1/2_ 289 μs) with an amplitude of 67%, an intermediate phase (t_1/2_ 3 ms) with an amplitude of 25%, and a slow phase (t_1/2_ 10 s) with an amplitude of 8%. The M246F strain displayed an extended half-time during the fast phase (t_1/2_ 366 μs [67%]) and an accelerated slow phase (6.7 s) with a slightly reduced amplitude compared to the control (Table 1). The M246A strain exhibited a more notable delay in the fast phase (t_1/2_ 451 μs), an increased amplitude at the intermediate phase (35%), and a retarded slow phase (t_1/2_ 13.4 s) (Fig. 7a, b and Table 1).

**Table 1:** Kinetic analysis of chlorophyll *a* fluorescence decay following a single saturating actinic flash in *Synechocystis* sp. PCC 6803 mutants with substitutions at Met246 and Asn250 of the D2 protein

		Fast Phase		Intermediate Phase		Slow Phase	
Strain	Treatment	Rate t_1/2_ (μs)	Amplitude (%)	Rate t_1/2_ (ms)	Amplitude (%)	Rate t_1/2_ (s)	Amplitude (%)
Control	No treatment	289 ± 28	67.3 ± 1.7	3.0 ± 0.3	25.0 ± 1.0	9.8 ± 1.6	7.7 ± 0.8
	DCMU			2.0 ± 0.1	9.8 ± 0.1	0.7 ± 0.1	90.2 ± 0.1
M246A	No treatment	451 ± 32	58.6 ± 3.4	3.6 ± 0.1	34.9 ± 1.7	13.4 ± 4.6	6.5 ± 0.9
	DCMU			1.5 ± 0.2	19.5 ± 0.5	1.7 ± 0.1	80.5 ± 0.5
M246F	No treatment	366 ± 62	66.6 ± 1.1	3.0 ± 0.1	27.8 ± 2.1	6.7 ± 0.5	5.6 ± 1.0
	DCMU			1.9 ± 0.4	8.1 ± 2.9	0.7 ± 0.1	91.9 ± 2.9
N250A	No treatment	350 ± 37	63.8 ± 1.8	5.0 ± 0.9	23.6 ± 2.3	13.8 ± 1.7	12.5 ± 0.6
	DCMU			0.9 ± 0.2	22.9 ± 2.4	0.14 ± 0.1	77.1 ± 2.4
N250H	No treatment	323 ± 43	60.5 ± 1.3	3.3 ± 0.1	27.7 ± 1.8	9.6 ± 0.8	11.9 ± 0.5
	DCMU			0.9 ± 0.1	23.1 ± 0.1	0.18 ± 0.1	76.9 ± 0.1

The experiment was repeated three times and the standard error is shown.

In the presence of DCMU, inhibition of forward electron transfer leads to Q_A_^−^ reoxidation through charge recombination with the donor side, resulting in two phases: a milliseconds (ms) phase which arises from charge recombination events involving Y_Z_^•^ (the oxidized form of D1-Tyr161 between the OEC and P680), P680^+^, and Q_A_^**−**^ and a slow phase on the order of seconds (s) which predominantly reflects the reoxidation of Q_A_^**−**^ by charge recombination with the S_2_ state of the OEC (Vass et al., 1999). In the presence of DCMU, the control strain exhibited a ms phase (t_1/2_ 2 ms) with an amplitude of 10% and a slow (s) phase (t_1/2_ 0.7 s) with an amplitude of 90%. The M246F strain showed similar kinetics to the control. In contrast, the M246A strain showed a much larger contribution of the ms phase (19.5%) than the control. This suggests an enhanced back reaction between Q_A_^**−**^ and Y_Z_^•^ or P680^+^, which could be a consequence of a misassembled OEC (Fig. 7c, d and Table 1).

To further investigate the effects of mutations on charge recombination in PS II, thermoluminescence (TL) assays were conducted (Fig. 7e, f and Supplemental Fig. S2). Excitation of the control strain with a single flash yielded three TL bands: the B band at 29 °C, associated with S_2_Q_B_^**−**^/S_3_Q_B_^**−**^ recombination; the C band at ca. 50 °C, linked to charge recombination between Y_D_^•^Q_A_^**−**^; (Y_D_ is D2-Tyr160 (Demeter et al., 1993; Johnson et al., 1994)) and the Q band at 20.4 °C, associated with S_2_Q_A_^**−**^ recombination in the presence of DCMU (Cser and Vass, 2007). Relative to the control cells the B band exhibited an increased temperature in the M246A (31.7 °C) and M246F (32.1 °C) strains, indicating a higher energy requirement for charge recombination between Q_B_^**−**^ and the S_2_ state (Fig. 7e). However, in the presence of DCMU, the Q band for the M246F strain was similar to the control, while the M246A strain (23.5 °C) exhibited a shift of ca. 3 °C and lacked the C band (Table 2 and Fig. 7f).

**Table 2:** Thermoluminescence temperature maxima for the Q-bands, B-bands and C-bands in *Synechocystis* sp. PCC 6803 mutants with substitutions at Met246 and Asn250 of the D2 protein

**Strain**	**Q band (°C)**	**B band (°C)**	**C band (°C)**
Control	20.4 ± 0.2	29.0 ± 1.0	53.4 ± 0.6
M246A	23.5 ± 1.2	31.7 ± 2.4	
M246F	20.6 ± 0.8	32.1 ± 1.5	
N250A	7.2 ± 0.2	27.2 ± 0.8	50.4 ± 1.6
N250H	9.1 ± 0.7	35.5 ± 0.3	47.8 ± 0.5

The experiment was repeated three times and the standard error is shown.

### Decay of variable chlorophyll *a* fluorescence and thermoluminescence in mutants with substitutions at Asn250

**Figure 8:**
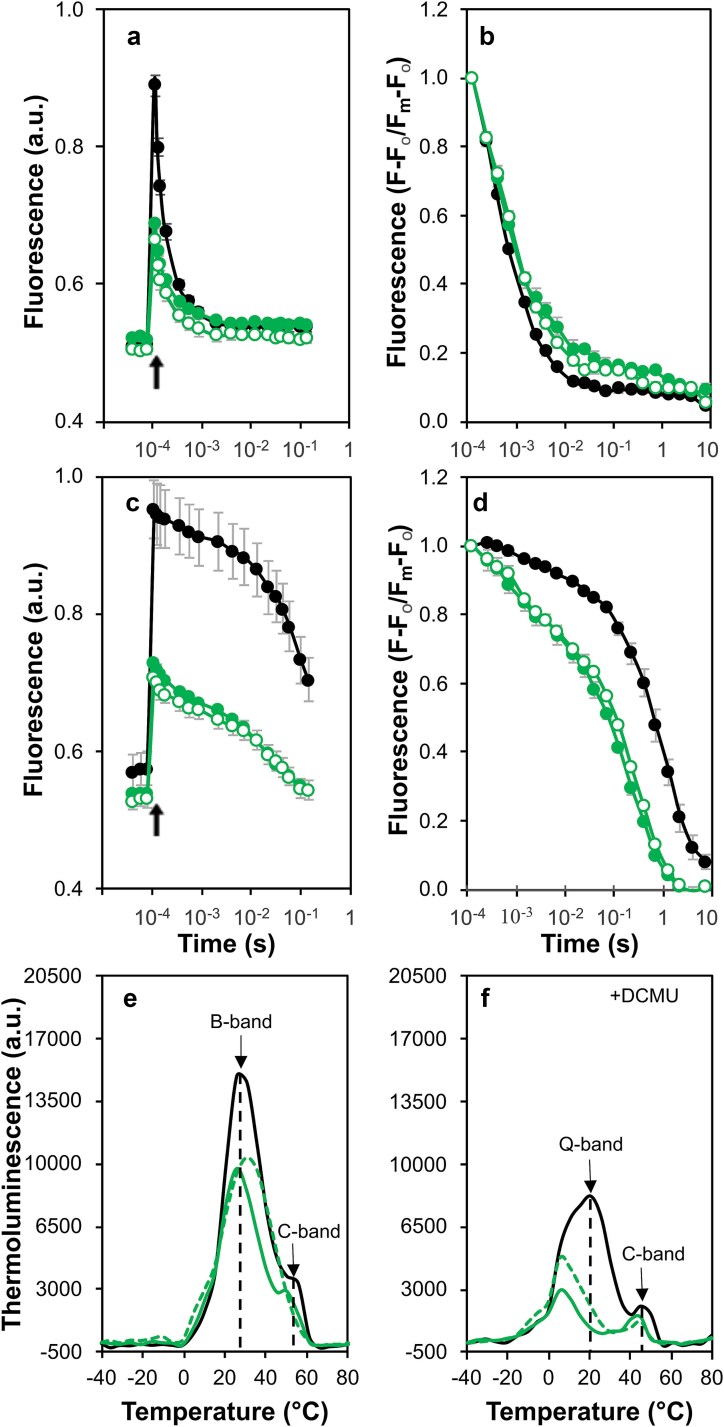
Chlorophyll *a* fluorescence decay after a single turnover actinic flash and thermoluminescence emission in the strains with mutations at Asn250. (a) Fluorescence decay after three measurements determining the initial (or F_o_) fluorescence. Control (black filled circles), N250A (green filled circles) and N250H (green empty circles). (b) Fluorescence decays from panel a normalized to the maximum fluorescence (Fm) where F is the fluorescence at each time point after the actinic flash. (c) Fluorescence decay after three measurements determining F_o_ in the presence of DCMU. Strains and symbols are the same as panel a. (d) Fluorescence decays from panel c normalized to Fm. In panels a–d, data are the average of three independent experiments. (e) Thermoluminescence emission in the absence of DCMU. Control (black line), N250A (green line), and N250H (green dashed lines). (f) Thermoluminescence emission in the presence of DCMU. Strains are the same as panel e. In panels e and f, the data are the average of three independent biological repeats. All thermoluminescence curves were deconvoluted (Supplementary Fig. S2) and the temperature maxima of the individual peaks are listed in Table 2.

Relative to control cells, the decay of the fast phase of the chlorophyll fluorescence after a single actinic flash in the N250A and N250H strains was slowed (350 μs and 323 μs, respectively) and was found to have a reduced amplitude (64% and 61%, respectively). Consequently, while the ms phase remained similar to the control in both strains, an increased amplitude (13% and 12%, respectively) was observed for the slow phase (Fig. 8a, b and Table 1). Notably, in the presence of DCMU, the fluorescence decayed more rapidly in the N250A and N250H strains than in control cells (Fig. 8c, d). Consequently, the amplitude of ms phase of the decay for the mutants increased from ~ 10% in the control to 23% in both strains (Table 1).

The accelerated decay of the chlorophyll fluorescence in the presence of DCMU was consistent with the TL data. While the B band in the N250A (27 °C) was slightly lower than the control (29 °C) that of the N250H was more stable (35.5 °C) (Fig. 8e). In contrast, the Q band was down shifted from 21 °C in the control to 7 °C in N250A cells and 9 °C in the N250H strain, consistent with a more negative redox potential for the Q_A_/Q_A_^−^ couple. Additionally, shifts at the C band were noted in the N250A mutant (50 °C) compared with 53 °C for the C band in control cells, while in the N250H mutant the C band was seen at 48 °C (Table 2 and Fig. 8f).

### PS II activity in the presence of bicarbonate and formate in mutants with substitutions at Met246

The D2 DE loop contributes directly to binding the bicarbonate cofactor to the non-heme iron of PS II (Fig. 1). The absence of the bicarbonate in PS II inhibits electron transfer from Q_A_ to Q_B_, a process reversed upon bicarbonate repletion (Govindjee et al., 1976; Eaton-Rye and Govindjee, 1988a; Eaton-Rye and Govindjee 1988b). Similarly, the addition of sodium formate (HCO_2_^−^) can displace bicarbonate, impairing Q_A_ to Q_B_ transfer (Robinson et al., 1984; Govindjee et al., 1991; Sedoud et al., 2011: Forsman et al., 2019; Forsman and Eaton-Rye, 2020). Since both Met246 and Asn250 are located within the ^240^AEETYSMVTAN^250^ sequence contributing to the bicarbonate-binding environment, the decay of chlorophyll *a* fluorescence following separate and combined addition of bicarbonate and formate was measured to investigate the binding state of bicarbonate and, by extension, forward electron transfer and putative protonation of Q_B_ in the mutants (Hussein et al., 2024).

**Figure 9:**
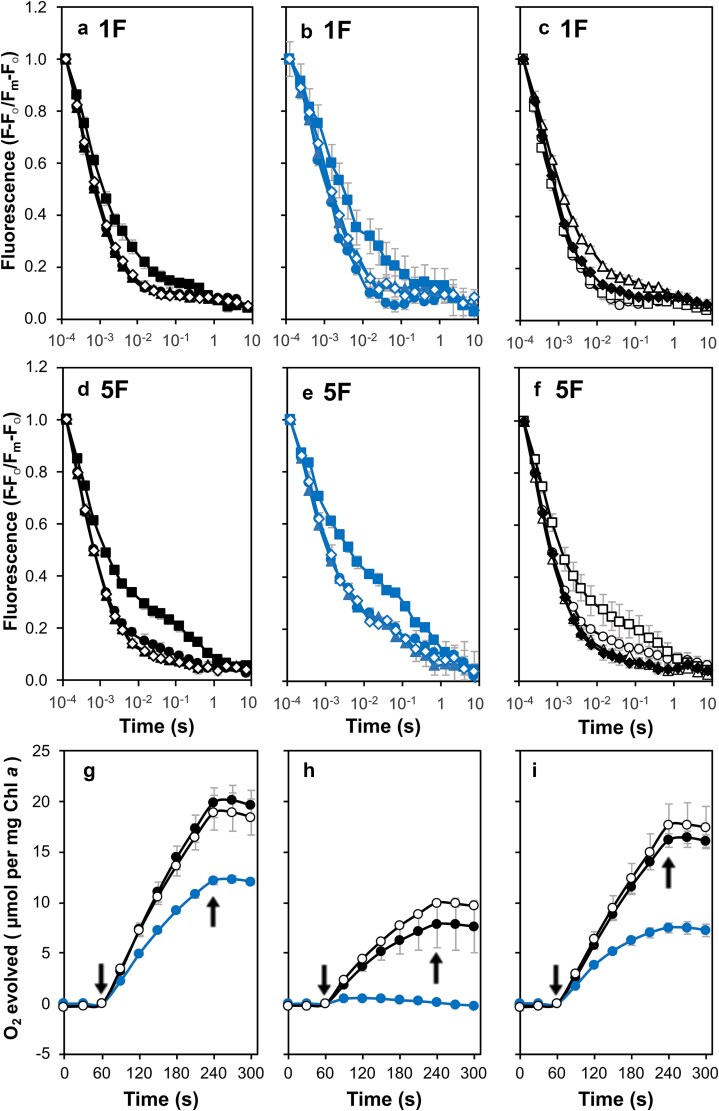
Chlorophyll *a* fluorescence decay and oxygen evolution in the presence of bicarbonate, formate, or bicarbonate and formate, in strains with mutations at Met246. (a) Fluorescence decay after one actinic flash in control cells. No treatment (black filled circles), 15 mM bicarbonate (black filled triangles), 25 mM formate (black filled squares) and 15 mM bicarbonate and 25 mM formate (black empty diamonds). (b) Fluorescence decay after one actinic flash in M246A cells. No treatment (blue filled circles), 15 mM bicarbonate (blue filled triangles), 25 mM formate (blue filled squares) and 15 mM bicarbonate and 25 mM formate (blue empty diamonds). (c) Fluorescence decay after one actinic flash in M246F cells. No treatment (black empty circles), 15 mM bicarbonate (black empty triangles), 25 mM formate (black empty squares) and 15 mM bicarbonate and 25 mM formate (black filled diamonds). (d) Fluorescence decay after five actinic flashes given at 5 Hz in control cells. Symbols as in panel a. (e) Fluorescence decay after five actinic flashes given at 5 Hz in M246A cells. Symbols as in panel b. (f) Fluorescence decay after five actinic flashes given at 5 Hz in M246F cells. Symbols as in panel c. (g) Oxygen evolution in the presence of 15 mM bicarbonate and 200 μM DMBQ. Control (black filled circles), M246A (blue filled circles) and M246F (black empty circles). (h) Oxygen evolution in the presence of 25 mM formate and 200 μM DMBQ. Strain identification as in panel g. (i) Oxygen evolution in the presence of 15 mM bicarbonate, 25 mM formate and 200 μM DMBQ. Strain identification as in panel g. In all assays of oxygen evolution, 1 mM K_3_Fe(CN)_6_ was added together with DMBQ. Arrows indicate actinic light on and off. All data shown in this figure are the average of three independent experiments.

In the presence of bicarbonate, the decay kinetics of the control strain after a single turnover were similar compared to untreated conditions (Fig. 9a). Similarly, the M246F strain exhibited similar decay rates in the presence of bicarbonate; however, after a single flash, the addition of bicarbonate in the M246A mutant was accompanied by a shift in the slow component from a t_1/2_ of 13.4 s (6.5%) to a t_1/2_ of 4.9 s (12.1%) (Fig. 9b, c and Supplementary Table S3). In addition, in the presence of formate, the fluorescence decay was impaired in all strains, and this was particularly pronounced in the M246A mutant. However, the effect of formate was reversible, as decay rates were restored with the addition of bicarbonate in all strains (Fig. 9a-c and Supplementary Table S3). Furthermore, exposure to five actinic flashes resulted in a noticeable delay in fluorescence decay in all treatments in the M246A strain (Fig. 9e-f). This suggests that the formation of Q_B_H_2_ and its exchange with PQ were slowed in the M246A mutant. This was not observed in the control and M246F strains.

The effect of formate addition and the combined addition of bicarbonate was also investigated for oxygen evolution supported by DMBQ. The presence of both DMBQ and bicarbonate increased oxygen evolution rates in the M264A and M246F strains compared to when DMBQ was present alone (cf. Fig. 4c, Fig. 9g, and Supplementary Table S1). Additionally, a decrease in oxygen evolution was observed in all strains when formate was added, with the M246A strain exhibiting virtually no oxygen evolution (Fig. 9h). The effects of formate, however, were reversed upon addition of bicarbonate (Fig. 9i and Supplementary Table S1).

### PS II activity in the presence of bicarbonate and formate in mutants with substitutions at Asn250

**Figure 10:**
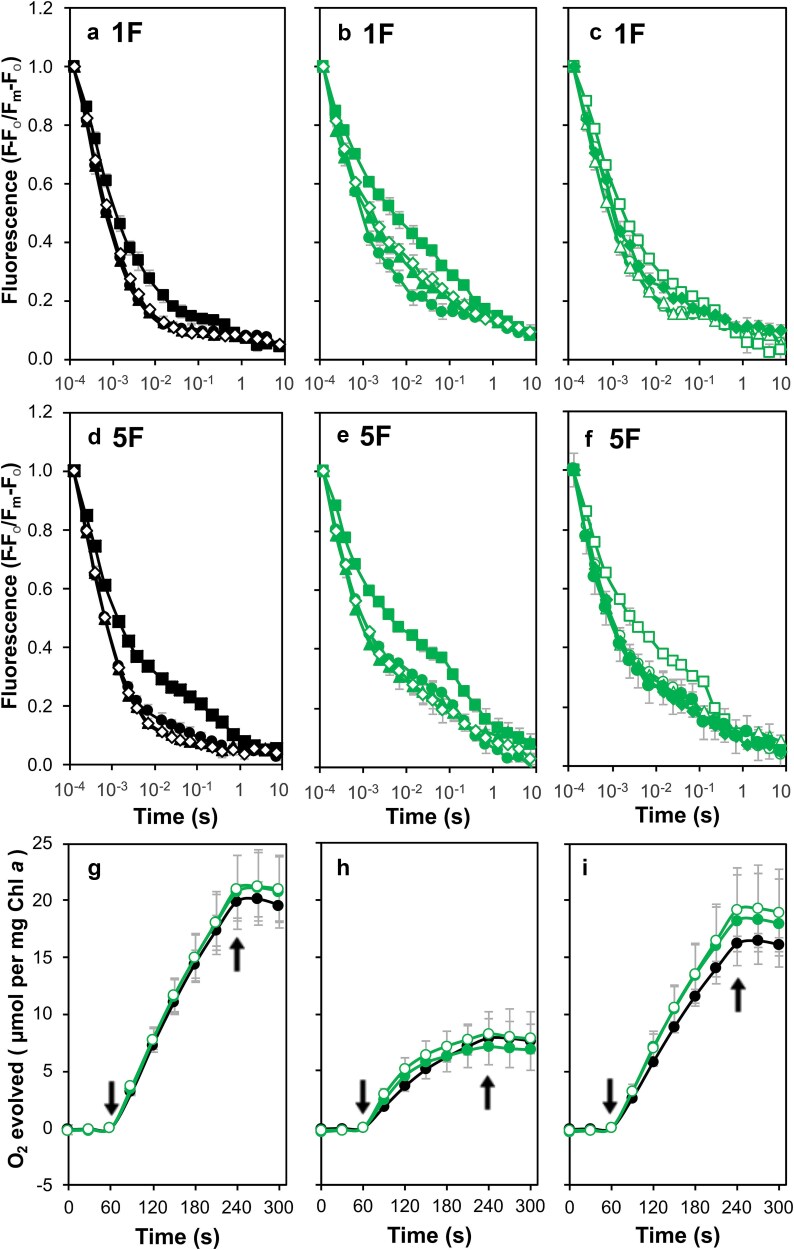
Chlorophyll *a* fluorescence decay and oxygen evolution in the presence of bicarbonate, formate, or bicarbonate and formate, in strains with mutations at Asn250. (a) Fluorescence decay after one actinic flash in control cells. These data are the same as Fig. 9a and are shown to aid comparison with the N250A and N250H mutants. No treatment (black filled circles), 15 mM bicarbonate (black filled triangles), 25 mM formate (black filled squares) and 15 mM bicarbonate and 25 mM formate (black empty diamonds). (b) Fluorescence decay after one actinic flash in N250A cells. No treatment (green filled circles), 15 mM bicarbonate (green filled triangles), 25 mM formate (green filled squares) and 15 mM bicarbonate and 25 mM formate (green empty diamonds). (c) Fluorescence decay after one actinic flash in N250H cells. No treatment (green empty circles), 15 mM bicarbonate (green empty triangles), 25 mM formate (green empty squares) and 15 mM bicarbonate and 25 mM formate (green filled diamonds). (d) Fluorescence decay after five actinic flashes given at 5 Hz in control cells. Symbols as in panel a. (e) Fluorescence decay after five actinic flashes given at 5 Hz in N250A cells. Symbols as in panel b. (f) Fluorescence decay after five actinic flashes given at 5 Hz in N250H cells. Symbols as in panel c. (g) Oxygen evolution in the presence of 15 mM bicarbonate and 200 μM DMBQ. Control (black filled circles), N250A (green filled circles) and N250H (green empty circles). (h) Oxygen evolution in the presence of 25 mM formate and 200 μM DMBQ. Strain identification as in panel g. (i) Oxygen evolution in the presence of 15 mM bicarbonate, 25 mM formate and 200 μM DMBQ. Strain identification as in panel g. In all assays of oxygen evolution, 1 mM K_3_Fe(CN)_6_ was added together with DMBQ. Arrows indicate actinic light on and off. All data shown in this figure are the average of three independent experiments.

When treated with bicarbonate, the N250A strain exhibited an accelerated fast phase (302 μs [65%] versus 350 μs [64%]) and a similar but less pronounced effect was seen in the N250H mutant (314 μs, [62%] versus 323 μs [61%]). In addition, in the N250A cells, a slowed slow phase (16.9 s [10.8%]) relative to untreated conditions (13.8 s [12.5%]) was observed (Supplementary Table S4). The slow phase of the N250H mutant, however, was similar in the presence and absence of bicarbonate. In contrast, in the presence of formate, the fast phase slowed in N250A and N250H cells (553 μs [51%] and 542 μs [50%], respectively); and an accelerated slow phase (1.9 s [18.3%] and 1.1 s [22.1%], respectively) was also observed compared to untreated cells and the slow component of the formate-treated control cells (6.6 s [11.1%]) (Fig. 10a-c and Supplementary Table S4). Following five actinic flashes, an additional delay in the decay was observed, in both N250A and N250H cells (Fig. 10d-f). These data are consistent with impaired formation or exchange of PQH_2_ in these mutants.

As seen with the M246A and M246F strains, the presence of both DMBQ and bicarbonate increased oxygen evolution rates in the N250A and N250H strains compared to when DMBQ was present alone (cf. Fig. 5c, Fig. 10g, and Supplementary Table S2). Addition of formate, however, slowed oxygen evolution in all strains in contrast to the extent of inhibition observed in M246A cells but again the addition of bicarbonate to the assay was able to reverse the formate effect (Fig. 10h, i and Supplementary Table S2).

### Spontaneous mutations in the N250A mutant

During this study, the N250A strain underwent two reversion events to the wild-type genotype while growing photoheterotrophically on solid agar, with one instance also featuring a CP43-Arg122 to Cys mutation. However, the phenotype of the D2-N250A:CP43-R122C double mutant was similar to the N250A strain. A CP43-R122C strain was then made independently and the phenotype was similar to the control strain, likewise the CP43-R122A and CP43-R122K mutants grew similarly to the control cells (Supplementary Fig. S3).

## Discussion

### The Met246 and Asn250 residues are found in proximity to the Q_A_-binding site

The DE loops of D1 and D2 participate in the assembly of the quinone-iron acceptor complex of PS II as well as being essential for supporting forward electron flow leading to the formation and exchange of plastoquinol (Zabret et al., 2021). In the RC47 complex, the D1 DE loop is displaced by the Psb28 assembly factor so neither bicarbonate nor Q_B_ are bound. In addition, the binding of D2-Glu241 to the NHI may play a role in preventing bicarbonate binding when Psb28 is present (Zabret et al., 2021; Xiao et al., 2021). It is hypothesized that, upon binding of CP43, Psb28 is released, and the DE loops reposition with D1-Tyr246 and D2-Tyr244 acting to stabilize ligation of bicarbonate to the NHI and D1-Phe265 acting to stabilize the binding of Q_B_ (Zabret et al., 2021; Brown et al., 2024) (Supplementary Fig. S4). Both D2-Met246 and D2-Asn250 are conserved in the motif surrounding D2-Tyr244 and participate in the hydrogen bond network that includes D2-Tyr244 and D2-Trp253, where D2-Trp253 is essential for the binding of Q_A_ (Vermaas et al., 1990). Given the conservation of D2-Met246 and D2-Asn250 (Supplemental Fig. S1) and their proximity to Q_A_ (Fig. 1), we investigated the role of these residues.

### The introduction of charged substitutions at Met246 and Asn250 prevented stable PS II assembly and activity

Mutations introduced at Met246 resulted in phenotypes that depended upon the introduced amino acid. Substitution with Phe in the M246F strain, preserving the hydrophobic character, resulted in cells with similar levels of PS II to the control; in contrast, removal of the bulky sidechain (in M246A cells) or introduction of a positive charge (in M246K cells) disrupted assembly with no detectable level of PS II observed in the M246K mutant (Fig. 2a–c and Fig. 3a–d). In addition, PS II activity, supported by the PS II-specific electron acceptor DMBQ also reflected the apparent level of PS II in the different mutants (Fig. 4a–c). Likewise, the phenotype obtained following the introduction of substitutions at Asn250 depended on the properties of the introduced amino acid. The introduction of Ala and His produced similar phenotypes with impaired photoautotrophic growth, reduced PS II assembly and reduced PS II activity; however, the introduction of a negative charge in the N250D mutant prevented stable assembly (Fig. 2d–f; Fig. 3e–h and Fig. 5a–c). The block on PS II assembly likely arises because of impaired Q_A_ binding as suggested for the D2-W253L mutant (Vermaas et al., 1990) (Fig. 1).

### Different responses to high light in the M246A and N250A mutants

The M246A mutant was susceptible to photodamage and exhibited impaired recovery under low light (Fig. 6a). A similar sensitivity was observed in the N250A strain (and the N250H mutant) but recovery was more extensive (Fig. 6c). The phenotype of the M246A cells resembled that of the D2-Y244A mutant which also exhibited slowed photoautotrophic growth and impaired recovery following exposure to high light (Khaing et al., 2022). D2-Met246 is within 3.4 Å of D2-Tyr244 and molecular dynamic simulations suggest D2-Tyr244 serves as a hydrogen bond donor to D2-Met246 (Sirohiwal and Pantazis, 2022). In addition, the inhibited rates of oxygen evolution following exposure to high light (Fig. 6a) resemble the phenotype of the D2-K264E mutant (Met264 is ~3.9 Å from Lys264) (Khaing and Eaton-Rye, 2023) (Fig. 1). D2-Lys264 also participates in the hydrogen-bond network that stabilizes bicarbonate binding and influences assembly; in addition, it appears to be involved in the accessibility of water and potentially CO_2_ (or HCO_3_^**−**^) to the NHI (Fantuzzi et al., 2022; Sugo et al., 2022; Sirohiwal and Pantazis, 2022). Destabilization of bicarbonate binding in the M246A cells would be consistent with the impaired assembly of PS II in this mutant (Fig. 3a–d and Fig. 4a, b), while the lack of recovery of the M246A cells under low light suggests Q_A_ may be released during the high-light exposure.

The persistence of impaired oxygen evolution in the M264A cells, when whole chain electron transport was measured under low light (Fig. 6b), contrasts with the phenotype of the N250A and N250H cells where sufficient centers are active in the presence of bicarbonate to support rates of oxygen evolution that remain essentially at Vmax throughout the high-light and low-light periods (Fig. 6d). This phenotype in the presence of bicarbonate was also observed in cells with mutations in the so-called PEST sequence (residues 226–233) of the DE loop of D1 (Nixon et al., 1995) as well as in cells lacking the PsbT LMW protein (Fagerlund et al., 2020). PsbT is situated at the monomer-monomer interface of the PS II dimer but the C-terminus of PsbT (PsbT-Pro27 and PsbT-Ile29) interact with D1-Phe239 and disruption of this interaction destabilizes bicarbonate binding to the NHI (Forsman and Eaton-Rye, 2021). Interestingly hydrogen bonds from Asn250 also interact with the C-terminus of PsbT, and Met246 comes within 3.9 Å of D1-Phe239 (Fig. 1). The above observations, combined with the presence of a hydrogen bond between Met246 and Asn250 (Fig. 1), led us to investigate the sensitivity of the mutants towards formate addition which is known to compete with bicarbonate binding at the NHI (Robinson et al., 1984; Sedoud et al., 2011).

### Modification of the bicarbonate and Q_A_ binding environments

Variable chlorophyll *a* fluorescence decay measurements after a single actinic flash in the M246A strain were slightly slowed relative to control cells (Fig. 7b and Table 1); but upon addition of formate the slow component was accelerated nine-fold with a three-fold increase in amplitude, consistent with a shift in the equilibrium for the sharing of an electron between Q_A_ and Q_B_ towards Q_A_^**−**^. The addition of formate also increased the millisecond component suggesting altered binding of Q_B_ but this putative effect on Q_B_ binding was similar to that seen in control cells (Fig. 9a, b and Supplementary Table S3). These effects were reversed upon bicarbonate addition. Furthermore, oxygen evolution to DMBQ was completely blocked by formate addition but likewise recovered upon bicarbonate addition (Fig. 9g-i).

The N250A (and N250H) cells also showed a sensitivity to formate addition with a similar shift in the apparent equilibrium constant for a sharing of the electron between Q_A_ and Q_B_ (Fig. 10a–c and Supplementary Table S4). In contrast to the M246A cells, however, PS II-specific oxygen evolution supported by DMBQ was not blocked when formate was added (Fig. 10g–i). A further difference between the M246A cells and the N250A and N250H mutants was apparent when the fluorescence decay was measured after a series of five single turnover actinic flashes suggesting that plastoquinone exchange is less efficient in the strains with mutations at Asn250 (cf. Fig. 9d–f and Fig. 10d–f). The mutants also behaved differently when the decay of fluorescence was measured in the presence of DCMU. In M246A cells the faster ms component, indicative of recombination with Y_Z_^•^ (or P680^+^) was increased suggesting that the assembly of the OEC may be perturbed in these cells (Fig. 7c, d). However, in the N250A and N250H cells, the back reaction was considerably accelerated relative to the control (Fig. 8c, d and Table 1). The modified back reaction kinetics led us to probe the apparent redox midpoint potentials of Q_A_/Q_A_^**−**^ and Q_B_/Q_B_^**−**^ through TL measurements which revealed a substantial decrease in the Q-band peak position of the N250A and N250H strains (Fig. 8e, f) consistent with a down shift in the Q_A_/Q_A_^**−**^ couple in agreement with the accelerated fluorescence decay in the presence of DCMU in these cells.

## Conclusion

The conservation of Met246 and Asn250 in the DE loop of D2 is consistent with their indispensable role in the binding environment of Q_A_ and bicarbonate in the quinone-iron acceptor complex. At both positions, the introduction of charged substitutions prevented PS II assembly. In contrast, the M246A, N250A, and N250H mutants were photoautotrophic although they differed in their responses to high-light exposure. The M246A cells were irreversibly inhibited by high light, possibly because of destabilized Q_A_ binding (Vass et al., 1992), whereas the N250A and N250H mutants recovered during a subsequent low-light period. The N250A and N250H mutants also exhibited a down shift in the mid-point potential the of Q_A_/Q_A_^**−**^ couple which resulted in an accelerated back reaction with the donor side of PS II. The increased probability of recombination in these cells, via the back reaction involving pheophytin, would increase the probability of the formation of ^1^O_2_ (Rutherford and Krieger-Liszkay, 2001; Vass, 2012) suggesting Asn250 plays a role in tuning the midpoint potential of Q_A_/Q_A_^**−**^ to favor forward electron transfer.

## Materials and Methods

### Strains and culture conditions

The *Synechocystis* 6803 glucose-tolerant substrain GT-O1 was used throughout this project (Williams, 1988; Morris et al., 2014). The D2 protein is encoded by two genes: *psbDI*, situated within the *psbDI:psbC* operon, and *psbDII* (Golden et al., 1989). The control strain retained an unmodified sequence while the modified sequences in the mutants were generated using a QuikChange II site-directed mutagenesis kit (Agilent, Santa Clara, CA, USA) with the primers listed in Supplementary Table S5. Six strains were generated (M246A, M246F, M246K, N250A, N250D, and N250H) by introducing plasmids carrying the modified *psbDI:psbC* operon into a deletion strain that lacked both *psbDII* and the *psbDI:psbC* operon (Khaing et al., 2020). In this system, the *psbDII* gene was substituted with a kanamycin-resistance cassette, while the *psbDI/C* operon was replaced by a chloramphenicol-resistance cassette. To facilitate the introduction of mutations, a spectinomycin-resistance cassette was positioned downstream of *psbC* to select for the insertion of the modified *psbDI:psbC* operon. Genotype and complete segregation were confirmed using colony PCR and Sanger sequencing.

All strains were maintained on BG-11 media agar plates supplemented with 5 mM glucose, 20 μM atrazine, 10 mM TES-NaOH (pH 8.2) and 0.3% sodium thiosulfate. Liquid cultures were grown mixotrophically in BG-11 medium containing 5 mM glucose. Both solid and liquid media contained appropriate antibiotics. Chloramphenicol was added at a concentration of 15 μg ml^−1^, while kanamycin and spectinomycin were added at 25 μg ml^−1^. Cultures were maintained at a light intensity of 30 μmol photons m^−2^ s^−1^ and a temperature of 30 °C. Photoautotrophic growth measurements were conducted under the same light and temperature conditions without glucose (Eaton-Rye, 2011).

### Oxygen evolution and photodamage assays

Oxygen evolution was assessed using a Clark-type oxygen electrode (Hansatech, King’s Lynn, U.K.) maintained at 30 °C. Samples, with a chlorophyll *a* concentration of 10 μg ml^−1^, were examined in the presence of 0.2 mM DMBQ, supplemented with 1 mM potassium ferricyanide (K_3_Fe(CN)_6_) or 15 mM sodium bicarbonate or 25 mM sodium formate. Saturating illumination was provided by a red light at an intensity of 2000 μmol photons m^−2^ s^−1^ from an FLS1 light source (Hansatech, UK) equipped with a 580 nm bandpass sharp cut-off glass filter (OG 580, Melles-Griot, Carlsbad, CA, USA).

High-light treatment to induce photodamage was achieved by exposing samples to 2000 μmol photons m^−2^ s^−1^ for 45 min using an Ektalite slide projector (Kodak, Rochester, NY, USA). Subsequently, the light was turned off, and the cultures were returned to standard conditions (30 μmol photons m^−2^ s^−1^) for an additional 135 min to allow for the recovery of PS II. Measurements were taken every 15 min for a total of 180 min in the presence of DMBQ (with K_3_Fe(CN)_6_) or 15 mM sodium bicarbonate.

### Low-temperature (77 K) fluorescence emission spectra

Fluorescence emission at 77 K was measured using a modified MPF-3 L fluorescence spectrometer (PerkinElmer, Waltham, MA, USA) fitted with a custom-made Dewar. Samples containing 5 μg ml^−1^ chlorophyll *a* were snap-frozen, and the spectra were collected after excitation at either 440 nm or 580 nm. Spectra were normalized to the emission maximum of PS I at 725 nm (Jackson et al., 2014).

### Measurement of variable chlorophyll *a* fluorescence

Variable chlorophyll *a* fluorescence induction and decay kinetics were measured using an FL-3500 fluorometer (PS I Instruments, Brno, Czech Republic). Samples were measured at a concentration of 5 μg ml^−1^ chlorophyll *a*. In fluorescence induction measurements, cells were dark adapted for 5 min and then subjected to measurement using a blue measuring flash (455 nm), with or without the presence of 40 μM DCMU. For the fluorescence decay experiments samples were dark adapted for 5 min and fluorescence was measured after a single-turnover actinic flashes (455 nm) or after a train of flashes spaced at 200 ms (5 Hz). Kinetic analyses were conducted following the procedures described by Vass et al. (1999). When present, sodium bicarbonate was at 15 mM, sodium formate 25 mM, and DCMU 40 μM.

### Thermoluminescence

Thermoluminescence measurements were conducted following the protocol outlined by Cser and Vass (2007). Cells equivalent to 30 μg of chlorophyll *a* were exposed to a single actinic flash in darkness at −10 °C and then further cooled to −40 °C. Thermoluminescence signals were recorded with a heating rate of 20 °C min^−1^ to 80 °C. When present, 60 μM DCMU was added to the sample in the dark before administering the actinic flash.

### Isolation of thylakoid membranes

Cells were grown to an OD of 0.8 and harvested in a cell wash solution containing 1 mM 6-amino caproic acid, 2 mM benzamidine, 20 mM CaCl_2_, 50 mM HEPES-NaOH (pH 7.5), 10 mM MgCl_2_ and 1 mM phenylmethylsulfonyl fluoride. The cells were then resuspended in a disruption buffer containing cell wash solution supplemented with 1 M betaine monohydrate and 800 mM sorbitol. This solution was then aliquoted into tubes containing 0.1 mm zirconia beads (BioSpec Products, Bartlesville, OK, USA). Cells were then mechanically disrupted in a bead beater (Biospec Products) for five cycles, each cycle consisting of 25 s at 4800 rpm followed by a 5 min incubation on ice. The glass beads and intact cells were removed by centrifugation at 8000 × g for 5 min. The thylakoid membranes were then harvested by centrifugation at 25 000 × g for 1 h at 4 °C, followed by resuspension in disruption buffer and a final centrifugation step at 25 000 × g for 25 min at 4 °C. The isolated thylakoids were snap-frozen in liquid nitrogen and stored at −80 °C in 25 mM BisTris-HCl (pH 7.0), 20% glycerol and 0.25 mg ml^−1^ Pefabloc (Sigma-Aldrich, St Louis, MO, USA).

### Blue native polyacrylamide gel electrophoresis and western blotting

Blue-native polyacrylamide gel electrophoresis (BN-PAGE) was conducted following the procedure outlined in Fagerlund et al. (2020). Thylakoid samples containing 1.2 μg chlorophyll *a* were loaded onto precast 4–16% Bis-Tris gradient gels (Life Technologies, Carlsbad, CA, USA) and electrophoresed at 4 °C. After completion of the gel run, proteins were transferred to a polyvinylidene difluoride membrane and subjected to probing with protein-specific antibodies (for D1, D2, and CP43 from Agrisera, Vännäs, Sweden). Anti-rabbit IgG peroxidase (Sigma-Aldrich) served as the secondary antibody, and visualization was achieved using enhanced chemiluminescence.

## Supplementary Material

Zhong_et_al_Supplementary_material_pcaf078

## Data Availability

Data are presented in this published article and its supporting information.
