# Supplementary material for: Met246 and Asn250 in the D2 protein are essential for the operation of the quinone-Fe-acceptor complex of Photosystem II
Source: Plant Cell Physiol. 2025 Jul 14;66(11):1730–49. doi: 10.1093/pcp/pcaf078 (PMC12661319; doi:10.1093/pcp/pcaf078)
Supplement: Zhong_et_al_Supplementary_material_pcaf078 [file zhong_et_al_supplementary_material_pcaf078.pdf]

Supplementary Material

Met246 and Asn250 in the D2 Protein are Essential for the Operation of the Quinone-Fe-Acceptor Complex of Photosystem II

Victor Zhong[1], Imre Vass[2], Priyanka Pradeep Patil[2,3] and Julian J. Eaton-Rye[1,*]

[1]Department of Biochemistry, University of Otago, P.O. Box 56, Dunedin 9054, New Zealand

[2]HUN-REN, Biological Research Center, Institute of Plant Biology, P.O. Box 521, Temesvári krt. 62, Szeged H-6726, Hungary

[3]Faculty of Science and Informatics, Doctoral School of Biology, University of Szeged, P.O. Box 652, Dóm tér 10, Szeged H-6720, Hungary

*Corresponding Author: Julian J. Eaton-Rye, Email, julian.eaton-rye@otago.ac.nz

Table of Contents

Page 3 Supplementary Fig. S1 Multiple protein sequence alignment of the D2 protein between Ala240 and Gly285 in selected cyanobacteria, algae and plants.

Page 4 Supplementary Fig. S2 Resolution of representative TL curves into individual components.

Page 5 Supplementary Fig. S3 Photoautotrophic growth measured by turbidity at 730 nm.

Page 6 Supplementary Fig. S4 Repositioning of the DE loop of D2 and selected residues of the DE Loop of D1 following the removal of the Psb28 assembly factor.

Page 7 Supplementary Table S1 Oxygen evolution rates in strains with mutations at Met246.

Page 8 Supplementary Table S2 Oxygen evolution rates in strains with mutations at Asn250.

Page 9 Supplementary Table S3 Kinetic analysis of chlorophyll *a* fluorescence decay following a single saturating actinic flash in strains with mutations at Met246.

Page 10 Supplementary Table S4 Kinetic analysis of chlorophyll *a* fluorescence decay following a single saturating actinic flash in strains with mutations at Asn250.

Page 11 Supplementary Table S5 Primers used for the introduction of targeted mutations into the *psbDI* gene using the QuikChange II site-directed mutagenesis kit.

Page 12 **References**

	222 232 242 252 262
Consensus	LFEDGEGANTFRAFNPTQAEETYSMVTANRFWSQIFGIAFS
Synechocystis sp. PCC 6803	LFEDGEDSNTFRAFEPTQAEETYSMVTANRFWSQIFGIAFS
Anabaena cylindrica PCC 7122	LFDDGEGSNTFPAFNPTQAEETYSMVTANRFWSQIFGIAFS
Calothrix sp. PCC 7507	LFKDTTGFNTFSGFTPTQAEETYSMVTANRYWSQIFGIAFS
Cyanothece sp. ATCC 51142	LFEDGEQANTFRAFEPTQAEETYSMVTANRFWSQIFGIAFS
Gloeobacter kilaueensis JS1	LFEDGEAPNTFKAFDPAQEEETYSMVLANRFWSQIFGIAFS
Nostoc sp. NIES-7120	LFEDGEGANTFRAFNPTQSEETYSMVTANRFWSQIFGIAFS
Synechocystis sp. PCC 6714	LFEDGEDSNTFRAFEPTQAEETYSMVTANRFWSQIFGIAFS
Synechococcus sp. PCC 7002	LFEDSDQANTFRAFEPTQAEETYSMVTANRFWSQIFGIAFS
Thermosynechococcus elongatus BP-1	LFQDGEGASTFRAFNPTQAEETYSMVTANRFWSQIFGIAFS
Thermosynechococcus vulcanus	LFQDGEGASTFRAFNPTQAEETYSMVTANRFWSQIFGIAFS
Tolypothrix tenuis PCC 7101	LFEDGEAANTFRAFNPTQSEETYSMVTANRFWSQIFGIAFS
Arabidopsis thaliana	LFEDGDGANTFRAFNPTQAEETYSMVTANRFWSQIFGVAFS
Chlamydomonas reinhardtii	LFEDGDGANTFRAFNPTQAEETYSMVTANRFWSQIFGVAFS
Nicotiana tabacum	LFEDGDGANTFRAFNPTQAEETYSMVTANRFWSQIFGVAFS
Pisum sativum	LFEDGDGANTFRAFNPTQAEETYSMVTANRFWSQIFGVAFS
Spinacia oleracea	LFEDGDGANTFRAFNPTQAEETYSMVTANRFWSQIFGVAFS

Supplementary Fig. S1 Multiple protein sequence alignment of the DE loop of the D2 protein in representative cyanobacteria, algae and plants. The location of the conserved Met246 and Asn250 residues within the bicarbonate-binding motif is shown in red. The bicarbonate-binding motif is shown in blue. The consensus sequence shows the most common anino acid at each position. The alignment was constructed using the Geneious platform.

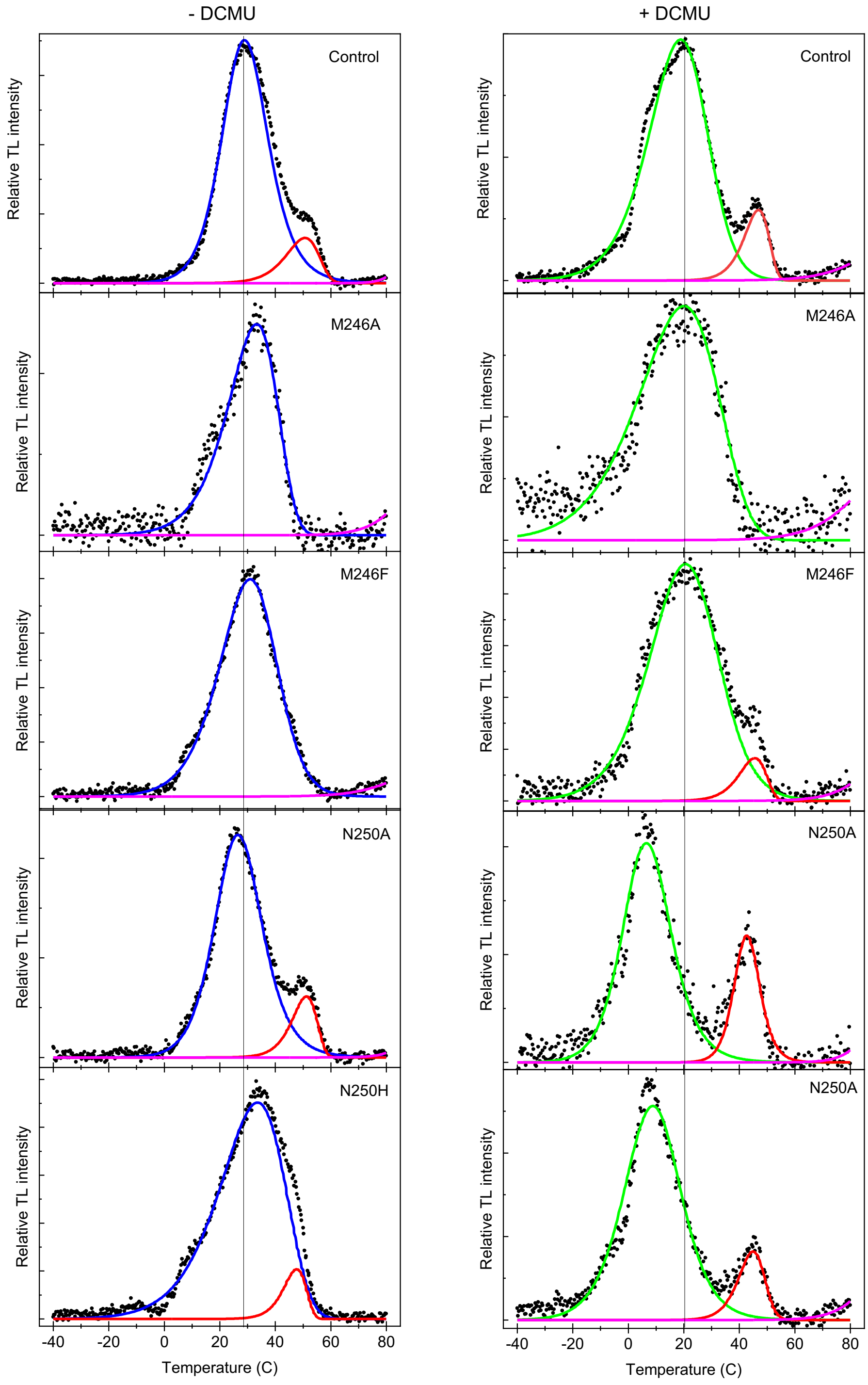

Supplementary Fig. S2 Resolution of representative thermoluminescence curves into individual components. Key: B-band (blue), Q-band (green), C-band (red), High temperature rise (pink). Fitting was performed according to Vass et al. (1981). The high temperature rise is unrelated to photosynthetic activity but is required for fitting (Hideg and Vass, 1993).

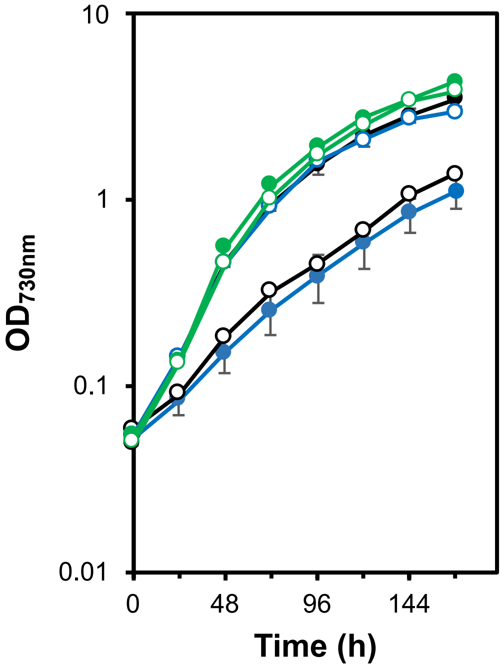

Supplementary Fig. S3 Photoautotrophic growth measured by turbidity at 730 nm. Control (black filled circles), N250A (blue filled circles), D2-N250A:CP43-R122C (black empty circles), CP43-R122A (blue empty circles), CP43-R122C (green filled circles) and CP43-R122K (green empty circles).

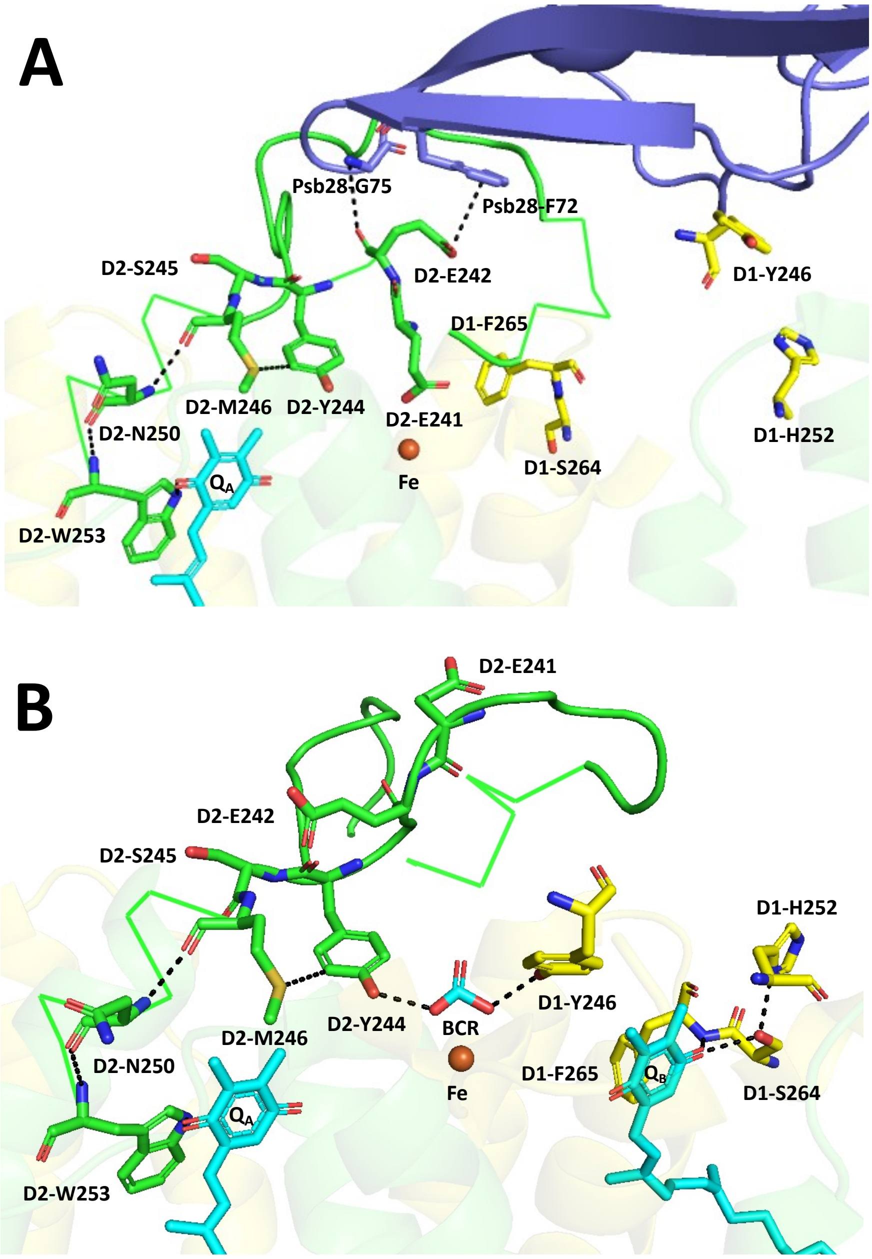

Supplementary Fig. S4 Repositioning of the DE loop of D2 and selected residues of the DE Loop of D1 following the removal of the Psb28 assembly factor. (A) The position of the D2 DE-Loop in the presence of Psb28. Data from PDB 7NHP (Zabret et al., 2021). (B) The position of the D2 DE-Loop after removal of Psb28 and the formation of the Q_B-binding environment containing D1-Phe265 and D1-Ser264. Large movements of D2-Glu241, D2-Glu242 and D1-Tyr246 together with the appearance of bicarbonate bound to the non-heme iron are also evident. Data from PDB 7N8O (Gisriel et al., 2022). Psb28 is in purple. D1 residues are in yellow and D2 residues in green. Q_A and Q_B (bound only after removal of Psb28) are in cyan. BCR, bicarbonate; Fe, non-heme iron. Oxygen atoms are in red and nitrogen atoms in blue. Selected hydrogen bonds are indicated by dashed lines.

Supplementary Table S1 Oxygen evolution rates in strains with mutations at Met246

Oxygen evolution rate (µmoles O_2 (mg of Chl)$^{-1}$ h^{-1})						
	Electron acceptor					
Strain	DCBQ	DMBQ	Bicarbonate	DMBQ + Bicarb.	DMBQ + Formate	DMBQ + Bicarb. + Formate
Control	539 ± 16	498 ± 7	538 ± 25	488 ± 49	204 ± 36	382 ± 26
M246A	278 ± 19	253 ± 5	487 ± 10	315 ± 8	6 ± 1	236 ± 20
M246F	650 ± 60	379 ± 12	573 ± 70	447 ± 41	239 ± 6	402 ± 54
M246K	0	0	15 ± 1	-	-	-

The standard error shown was calculated from the average of three independent experiments.

Supplementary Table S2 Oxygen evolution rates in strains with mutations at Asn250

Oxygen evolution rate (µmoles O_2 (mg of Chl)$^{-1}$ h^{-1})						
	Electron acceptor					
Strain	DCBQ	DMBQ	Bicarbonate	DMBQ + Bicarb.	DMBQ + Formate	DMBQ + Bicarb. + Formate
Control	539 ± 16	498 ± 7	538 ± 25	488 ± 49	204 ± 36	382 ± 26
N250A	472 ± 88	426 ± 46	596 ± 77	505 ± 65	227 ± 21	453 ± 108
N250D	0	0	24 ± 8	-	-	-
N250H	410 ± 13	445 ± 47	649 ± 8	485 ± 67	266 ± 47	446 ± 81

The standard error shown was calculated from the average of three independent experiments.

Supplementary Table S3 Kinetic analysis of chlorophyll *a* fluorescence decay following a single saturating actinic flash in strains with mutations at Met246

		Fast Phase		Intermediate Phase		Slow Phase	
Strain	Treatment	Rate $t_{1/2}$ (μs)	Amplitude (%)	Rate $t_{1/2}$ (ms)	Amplitude (%)	Rate $t_{1/2}$ (s)	Amplitude (%)
Control	No treatment	289 ± 28	67.3 ± 1.7	3.0 ± 0.3	25.0 ± 1.0	9.8 ± 1.6	7.7 ± 0.8
	Bicarbonate	306 ± 21	68.8 ± 0.1	3.4 ± 0.2	24.3 ± 0.4	7.2 ± 1.0	6.9 ± 0.3
	Formate	408 ± 24	66.2 ± 2.6	9.1 ± 0.5	22.7 ± 1.1	6.6 ± 0.5	11.1 ± 1.5
	Bicarb. + Formate	314 ± 34	67.4 ± 2.2	3.9 ± 0.3	25.1 ± 2.6	10.3 ± 2.4	7.5 ± 0.4
	DCMU			2.0 ± 0.1	9.8 ± 0.1	0.7 ± 0.1	90.2 ± 0.1
M246A	No treatment	451 ± 32	58.6 ± 3.4	3.6 ± 0.1	34.9 ± 1.7	13.4 ± 4.6	6.5 ± 0.9
	Bicarbonate	427 ± 40	56.4 ± 2.6	3.9 ± 0.5	31.5 ± 5.0	4.9 ± 0.6	12.1 ± 2.4
	Formate	590 ± 27	50.2 ± 3.0	10.3 ± 1.3	31.0 ± 2.5	1.5 ± 0.7	18.8 ± 0.9
	Bicarb. + Formate	421 ± 28	52.0 ± 0.9	3.7 ± 0.2	37.3 ± 0.9	9.0 ± 1.8	10.7 ± 1.7
	DCMU			1.5 ± 0.2	19.5 ± 0.5	1.7 ± 0.1	80.5 ± 0.5
M246F	No treatment	366 ± 62	66.6 ± 1.1	3.0 ± 0.1	27.8 ± 2.1	6.7 ± 0.5	5.6 ± 1.0
	Bicarbonate	293 ± 13	65.0 ± 1.9	2.9 ± 0.3	28.5 ± 2.1	3.0 ± 0.5	6.5 ± 0.2
	Formate	487 ± 20	64.3 ± 0.1	6.9 ± 2.2	25.7 ± 1.9	5.7 ± 1.3	10.0 ± 1.9
	Bicarb. + Formate	374 ± 42	67.7 ± 0.6	4.2 ± 0.4	23.4 ± 0.4	7.6 ± 1.7	8.9 ± 0.9
	DCMU			1.9 ± 0.4	8.1 ± 2.9	0.7 ± 0.1	91.9 ± 2.9

[a] The fast (μs) component represents forward electron transfer from Q_A^- to Q_B or, if present, Q_B^-. The intermediate (ms) component represents electron transfer from Q_A^- when the Q_B-binding site is empty and a Q_B from the plastoquinone pool must first bind to the vacant site before the electron is transferred. The slow (s) component represents the back reaction from Q_A^- to the Mn_4CaO_5 cluster in the S_2 state (the Mn_4CaO_5 cluster or oxygen-evolving complex cycles through five ($S_{0\text{-}4}$) oxidation states). The kinetics of the first two phases are exponential functions, whereas the slow phase is described by a hyperbolic function. Following Vass et al. (1999) the decay curve was therefore deconvoluted using the equation $F_{(t)} – F_o = A_1\exp^{(-t/T1)} + A_2\exp^{(-t/T2)} + A_3/(1 + t/T3)$; where, $F_{(t)}$ is the yield of variable fluorescence, F_o is the initial fluorescence from dark-adapted cells, A_1, A_2, A_3 are the amplitudes and T_1, T_2, T_3 are the time constants. The T_1 and T_2 values are used to calculate the half-time of the fast and intermediate phases by using the equation $t_{1/2} = \ln 2\ T$, whereas the half-time for hyperbolic phase is equal to T_3.

[b] In the presence of DCMU, the ms exponential component likely reflects recombination with $TyrZ^{\bullet}$ (or $P680^+$ if the Mn_4CaO_5 cluster is impaired) and the slow (s) hyperbolic component reflects recombination with the S_2 state of the Mn_4CaO_5 cluster (Vass et al. 1999).

The standard error is calculated from three biological repeats.

Supplementary Table S4 Kinetic analysis of chlorophyll *a* fluorescence decay following a single saturating actinic flash in strains with mutations at Asn250[a]

Strain	Treatment	Fast Phase		Intermediate Phase		Slow Phase	
		Rate $t_{1/2}$ (µs)	Amplitude (%)	Rate $t_{1/2}$ (ms)	Amplitude (%)	Rate $t_{1/2}$ (s)	Amplitude (%)
Control	No treatment	289 ± 28	67.3 ± 1.7	3.0 ± 0.3	25.0 ± 1.0	9.8 ± 1.6	7.7 ± 0.8
	Bicarbonate	306 ± 21	68.8 ± 0.1	3.4 ± 0.2	24.3 ± 0.4	7.2 ± 1.0	6.9 ± 0.3
	Formate	408 ± 24	66.2 ± 2.6	9.1 ± 0.5	22.7 ± 1.1	6.6 ± 0.5	11.1 ± 1.5
	Bicarb. + Formate	314 ± 34	67.4 ± 2.2	3.9 ± 0.3	25.1 ± 2.6	10.3 ± 2.4	7.5 ± 0.4
	DCMU			2.0 ± 0.1	9.8 ± 0.1	0.7 ± 0.1	90.2 ± 0.1
N250A	No treatment	350 ± 37	63.8 ± 1.8	5.0 ± 0.9	23.6 ± 2.3	13.8 ± 1.7	12.5 ± 0.6
	Bicarbonate	302 ± 21	65.2 ± 3.4	4.8 ± 0.5	24.0 ± 2.7	16.9 ± 3.7	10.8 ± 1.4
	Formate	553 ± 87	50.9 ± 2.9	9.9 ± 2.1	31.0 ± 1.7	1.9 ± 0.7	18.3 ± 1.7
	Bicarb. + Formate	353 ± 10	63.8 ± 2.6	5.4 ± 0.8	24.5 ± 3.0	13.5 ± 3.4	11.7 ± 0.4
	DCMU			0.9 ± 0.2	22.9 ± 2.4	0.14 ± 0.1	77.1 ± 2.4
N250H	No treatment	323 ± 43	60.5 ± 1.3	3.3 ± 0.1	27.7 ± 1.8	9.6 ± 0.8	11.9 ± 0.5
	Bicarbonate	314 ± 77	62.0 ± 0.7	4.0 ± 1.0	25.8 ± 0.2	11.0 ± 5.4	12.1 ± 0.5
	Formate	542 ± 92	50.0 ± 0.7	4.0 ± 0.8	27.3 ± 1.0	1.1 ± 0.2	22.1 ± 1.6
	Bicarb. + Formate	395 ± 42	65.1 ± 0.7	9.1 ± 2.6	21.9 ± 1.3	13.2 ± 1.5	13.0 ± 0.6
	DCMU			0.9 ± 0.1	23.1 ± 0.1	0.18 ± 0.1	76.9 ± 0.1

[a] The rates and amplitudes were calculated as described in Supplemental Table S3.

The standard error is calculated from three biological repeats.

Supplementary Table S5 Primers used for the introduction of targeted mutations into the *psbDI* gene using the QuikChange II site-directed mutagenesis kit

Primers used in QuikChange Site-Directed Mutagenesis	
D2-M246A-DS	AGCAGAAGAAACCTATTCCGCCGTGACCGCTAACCGTTTC
D2-M246A-US	GAAACGGTTAGCGGTCACGGCGGAATAGGTTTCTTCTGCT
D2-M246F-DS	GCAGAAGAAACCTATTCCTTCGTGACCGCTAACCGTTTC
D2-M246F-US	GAAACGGTTAGCGGTCACGAAGGAATAGGTTTCTTCTGC
D2-M246K-DS	CAAGCAGAAGAAACCTATTCCAAGGTGACCGCTAAC
D2-M246K-US	GTTAGCGGTCACCTTGGAATAGGTTTCTTCTGCTTG
D2-N250A-DS	CCTATTCCATGGTGACCGCTGCCCGTTTCTGGTCTCA
D2-N250A-US	TGAGACCAGAAACGGGCAGCGGTCACCATGGAATAGG
D2-N250D-DS	CTATTCCATGGTGACCGCTGACCGTTTCTGGTC
D2-N250D-US	GACCAGAAACGGTCAGCGGTCACCATGGAATAG
D2-N250H-DS	CTATTCCATGGTGACCGCTCACCGTTTCTGGTC
D2-N250H-US	GACCAGAAACGGTGAGCGGTCACCATGGAATAG

References

Hideg, É. And Vass, I. (1993) The 75°C Thermoluminescence band of green tissues: Chemiluminescence from membrane-chlorophyll interaction. *Photochem. Photobiol.* 58: 280–283. https://doi.org/10.1111/j.1751-1097.1993.tb09562.x

Gisriel, C.J., Wang, J., Liu, J., Flesher, D.A., Reiss, K.M., Huang, H.-L., Yang, K.R., Armstrong, W.H., Gunner, M., Batista, V.S., Debus, R.J. and Brudvig, G.W. (2022) High-resolution cryo-electron microscopy structure of Photosystem II from the mesophilic cyanobacterium, *Synechocystis* sp. PCC 6803. *Proc. Natl. Acad. Sci. USA* 119: e2116765118. https://doi.org/10.1073/pnas.2116765118

Vass, I, Horváth, G., Herczeg, T. and Demeter, S. (1981) Photosynthetic energy conservation investigated by thermoluminescence: Activation energies and half-lives of thermoluminescence bands of chloroplasts determined by mathematical resolution of glow curves. *Biochim. Biophys. Acta* 634: 140–152. https://doi.org/10.1016/0005-2728(81)90134-1

Vass, I., Kirilovsky, D. and Etienne, A.-L. (1999) UV-B radiation-induced donor-and acceptor-side modifications of Photosystem II in the cyanobacterium *Synechocystis* sp. PCC 6803. *Biochemistry* 38: 12786–12794. https://doi.org/10.1021/bi991094w

Zabret, J., Bohn, S., Schuller, S.K., Arnolds, O., Möller, M., Meier-Credo, J., Liauw, P., Chan, A., Tajkhorshid, E., Langer, J.D., Stoll, R., Krieger-Liszkay, A., Engel, B.D., Rudack, T., Schuller, J.M. and Nowaczyk, M.M. (2021) Structural insights into Photosystem II assembly. *Nat. Plants* 7: 524–538. https://doi.org/10.1038/s41477-021-00895-0
